# HDAC7 acts as an astrocytic mediator of Aβ pathology that directly engages IKK to drive astrocyte neurotoxicity and neurodegeneration in Alzheimer’s disease

**DOI:** 10.1186/s13195-026-02121-5

**Published:** 2026-06-22

**Authors:** Jinwang Ye, Yunsong Deng, Bingge Zhang, Chengjia Li, Xing Guo, Ruizhu Yue, Huali Wan, Yue Hao, Shifeng Xiao

**Affiliations:** 1 Brain Disease and Big Data Research Institute, College of Life Sciences and Oceanography, Shenzhen University, Shenzhen, 518060 Guangdong China; 2 Shenzhen-Hong Kong Institute of Brain Science-Shenzhen Fundamental Research Institutions, Shenzhen, 518055 China; 3 Department of Pathophysiology, School of Basic Medicine, Key Laboratory of Ministry of Education of China and Hubei Province for Neurological Disorders, Tongji Medical College, Huazhong University of Science and Technology, Wuhan, 430030 China; 4 Department of Laboratory Medicine, The First Affiliated Hospital of Shenzhen University, Shenzhen Second People’s Hospital, Shenzhen, 518035 China; 5 School of Pharmacy, Shenzhen University Medical School, Shenzhen University, Shenzhen, 518055 China

**Keywords:** HDAC7, NF-κB, Amyloid‐β, Reactive astrocyte, Neurodegeneration, Alzheimer’s disease

## Abstract

**Background:**

Astrocytes undergo reactive transformations in response to pathological stimuli and play a critical role in neuronal loss associated with Alzheimer’s disease (AD). However, the intrinsic mechanisms through which astrocytes detect amyloid-β (Aβ) pathology and develop neurotoxic properties remain inadequately understood. The dysregulation of class IIa Histone deacetylases (HDACs) has been implicated in astrocyte dysfunction under pathological conditions. This study aims to elucidate the role of HDAC7 as an astrocytic mediator of Aβ that drives the formation of neurotoxic reactive astrocytes, and to propose HDAC7 as a potential therapeutic target for mitigating neuronal loss and cognitive deficits in AD.

**Methods:**

We examined HDAC7 expression in APP/PS1 mice of varying ages using RT-qPCR, Western blotting, and immunostaining analysis. Astrocyte-specific HDAC7 overexpression and knockdown were achieved through adeno-associated virus (AAV) delivery (GfaABC1D promoter) in wild-type (WT) and APP/PS1 mice, followed by behavioral tests, immunostaining, RT-qPCR, and RNA-seq. Mechanistic studies were conducted using primary astrocytes derived from WT and *Hdac7*^*flx/flx*^ mice, employing co-immunoprecipitation, Western blotting, and neuron viability assays. Pharmacological inhibition of HDAC7 in APP/PS1 mice was performed via intraperitoneal injection of TMP195, and the effects on neurotoxic reactive astrocytes, neuronal and synaptic loss, and behavioral performance were measured.

**Results:**

HDAC7 was selectively upregulated in plaque-adjacent astrocytes in APP/PS1 mice. Overexpression of HDAC7 specifically in astrocytes was sufficient to induce a neurotoxic transcriptional profile, neuronal loss, and cognitive deficits in both WT and young APP/PS1 mice. Mechanistically, upon Aβ stimulation, the upregulated HDAC7 directly interacted with and deacetylated IKKα and IKKβ, resulting in the activation of IKK, translocation of NF-κB to the nucleus, and subsequent expression of neurotoxic genes. This neurotoxic conversion was dependent on IKK activity, as IKK inhibition nullified the effects in astrocytes overexpressing HDAC7. Conversely, astrocytic HDAC7 knockdown or treatment with TMP195 attenuated IKK-NF-κB signaling, reduced the presence of neurotoxic reactive astrocytes, and rescued neurodegeneration and cognitive deficits in APP/PS1 mice.

**Conclusions:**

HDAC7 acts as an intrinsic effector within astrocytes, responding to Aβ pathology and converting astrocytes into a neurotoxic state through direct interaction with IKK. Targeting HDAC7 presents a promising strategy for astrocyte-directed therapeutic interventions in Alzheimer’s disease.

**Supplementary Information:**

The online version contains supplementary material available at https://doi.org/10.1186/s13195-026-02121-5.

## Introduction

Astrocytes, the most abundant glial cell population in the central nervous system (CNS), are integral to maintaining brain homeostasis under both physiological and pathophysiological conditions. In the context of neuroinflammation, neural injury, or neurodegenerative disorders, astrocytes undergo significant morphological and molecular transformations, transitioning into a reactive state [1, 2]. These reactive astrocytes are heterogeneous, with various subpopulations identified across distinct neuroanatomical regions, neural circuits, and disease contexts [3–5]. Previous molecular profiling studies of reactive astrocytes in ischemic and neuroinflammatory mouse models have identified two identified two primary subtypes or substates: neurotoxic astrocytes (referred to as A1 astrocytes) and neuroprotective astrocytes (referred to as A2 astrocytes) [1, 6]. The cytokines interleukin (Il)−1α, tumor necrosis factor (TNF) α and complement component 1q (C1q) secreted by classically activated microglia are sufficient to induce neurotoxic reactive astrocytes *in vitro*. Neurotoxic reactive astrocytes often undergo pathological alterations that result in dysfunctional glia-neuron interactions, compromised synaptic transmission, and neuronal death [7, 8]. Postmortem analysis of human brain specimens has revealed the abundant prevalence of neurotoxic reactive astrocytes in individuals with neurodegenerative diseases, including Alzheimer’s disease (AD), Parkinson’s disease, and amyotrophic lateral sclerosis [6, 9, 10].

AD is the most prevalent neurodegenerative disease characterized with deposition of β-amyloid (Aβ) plaques and intracellular accumulation of tau neurofibrillary tangles (NFTs) as the primary hallmarks. Neurotoxic reactive astrocytes are ubiquitously present in postmortem AD brain tissues and AD mice models, and are closely associated with pathological tau burden, synaptic dysfunction and cognitive decline [11–13]. Blockage of neurotoxic reactive astrocytes has been shown to mitigate neuronal loss, neuroinflammation, and the pathogenic progression of neurodegeneration in AD and other neurodegenerative diseases [9, 10, 14]. In the 5xFAD mouse model, blockage of neurotoxic reactive astrocytes by potentiating the Nrf2 pathway mitigate neurodegeneration and rescued cognitive deficits [15]. In a tauopathy mouse model, blockage of astrocyte reactivity by a selective Na^+^/K^+^ ATPases inhibitor, digoxin, suppressed neuroinflammation and reduced brain atrophy [16]. Furthermore, neurotoxic reactive astrocytes have been observed to be closely surrounding Aβ plaques [17], suggest an intricate interactive link between neurotoxic reactive astrocytes and Aβ pathology. However, the underlying mechanisms and potential therapeutic targets that drives such astrocyte neurotoxicity in AD pathology have not been identified.

The formation of neurotoxic reactive astrocytes is orchestrated by a network of subtype-specific gene transcription, mediated by transcription factors, chromatin regulators, and modifying enzymes that facilitate the acquisition of neurotoxic functions while diminishing neuro-supportive capabilities [15, 18, 19]. Within this regulatory framework, histone deacetylases (HDACs) are compelling due to their roles in gene transcription and their potential as therapeutic targets for various neurological diseases [20, 21]. In mammals, there are 18 HDAC enzymes (HDAC1-11, SIRT1-7), with class IIa HDACs (HDAC4, 5, 7, 9) being particularly noteworthy due to their tissue-and cell-type- specific functions within the immune system [22–24]. These Enzymes regulates signaling transduction and gene expression through nuclear-cytoplasmic shuttling [25, 26]. Dysregulation or overexpression of class IIa HDACs has been implicated in astrocyte dysfunction in various neurological disorders [27, 28]. For example, upregulation of HDAC4 and HDAC7 is associated with impaired clearance function of astrocytes for Aβ and tau aggregates [29, 30]. Furthermore, inhibitors targeting class IIa HDACs have demonstrated efficacy in animal models of neuroinflammatory diseases. The underlying mechanisms likely involve multiple pathways, including the suppression of innate immune cell activation and the promotion of neuronal survival [31–33].

In this study, we aimed to elucidate the role of class IIa HDACs in the development of neurotoxic reactive astrocytes and the progression of neurodegeneration in AD. Our findings indicate that the upregulation of HDAC7 in astrocytes is correlated with the induction of neurotoxic reactive astrocytes, neuronal death, and cognitive impairments in APP/PS1 mice. We discovered a novel mechanism in which elevated HDAC7 levels activate the NF-κB signaling pathway via deacetylating IKKα and IKKβ, leading to the neurotoxic phenotype of reactive astrocytes. Furthermore, the knockdown of HDAC7 or its pharmacological inhibition effectively prevented the formation of peri-plaque neurotoxic reactive astrocytes, preserved neuronal and synaptic functions, and alleviated Aβ pathology and cognitive deficits in the APP/PS1 mouse model. These findings highlight the therapeutic potential of astrocyte-specific interventions targeting HDAC7 for the treatment of AD and related neurodegenerative diseases.

## Materials and methods

### Mice

The APP/PS1 (APPswe/PSEN1dE9, #034832) double‐transgenic mice were obtained from the Jackson Laboratory, and the wildtype C57BL/6 mice were from Changzhou Cavens Laboratory Animal Co., Ltd (Changzhou, China). The *Hdac7*^flx/flx^ mice (C57BL/6JGpt-Hdac7em1Cflox/Gpt, Strain NO. T069577) were procured from GemPharmatech Co., Ltd. The mice were housed in groups of four to five per cage, and were maintained under a 12 h light/dark cycle at temperatures 22 ± 2 °C. The animals were allowed free access to food and water. Both male and female mice were used in all experiments, with approximately equal representation of each sex in each experimental group. Age‐ and sex‐matched mice were randomly assigned to experimental groups. This study adhered to all relevant ethical regulations concerning animal testing and research. All animal experiments conducted in this study received approval from the Institutional Animal Care and Use Committee at Shenzhen University (Approval No. IACUC-202300171).

### Stereotaxic brain injection

For brain stereotactic injection, mice were anesthetized using isoflurane and subsequently positioned on a stereotaxic apparatus. The surgical site was sterilized with iodophor, followed by a midline incision along the scalp. Bilateral craniotomies were performed at coordinates posterior 2.1 mm and lateral ± 1.4 mm relative to the bregma. Using a microinjection system (World Precision Instruments), the following viral vectors were bilaterally administered into the hippocampal region at a rate of 50 nL/min: AAV-GfaABC1D-HDAC7-3xFlag (1.0 μL, 4.2 × 10^12^ vg/mL), AAV-GfaABC1D -GFP (1.0 μL, 5.0 × 10^12^ vg/mL), AAV-GfaABC1D-shHDAC7-GFP (1.0 μL, 4.6 × 10^12^ vg/mL) or AAV-GfaABC1D-Scramble-GFP (1.0 μL, 4.1 × 10^12^ vg/mL). The injection needle was retained in position for 5 min prior to withdrawal. Subsequently, the incision was sutured, and the mice were placed near a heat source to aid in recovery. For control groups, both WT and APP/PS1 mice received an equivalent volume of AAV-GfaABC1D-GFP (for HDAC7 overexpression experiments) or AAV-GfaABC1D-shScramble-GFP (for HDAC7 knockdown experiments).

### Drug administration

For intraperitoneal administration, TMP195 was prepared at a concentration of 7.5 mg/mL in sterile 0.9% saline, supplemented with 5% (v/v) Tween-80 and 20% (v/v) PEG-300. APP/PS1 mice received intraperitoneal injections of the prepared TMP195 (50 mg/kg) or vehicle every three days over a period of two months. Control mice received intraperitoneal injections of the same volume of vehicle (sterile 0.9% saline containing 5% Tween-80 and 20% PEG-300) following the same schedule. Following the treatment regimen, behavioral experiments were conducted. Subsequently, all mice were euthanized, and their hippocampal tissues were either homogenized or fixed for pathological analysis.

### Morris Water Maze (MWM) test

The MWM behavioral test was conducted to evaluate the spatial learning and memory capabilities of mice based on the previous study [34]. During the training phase, mice were subjected to daily training sessions in the water maze over the course of five consecutive days, with three trials conducted each day. These trials were scheduled between 14:00 and 20:00, with a 30-s inter-trial interval. Each trial required the mice to locate a submerged platform within a 60-s timeframe. Upon successfully reaching the platform, the trial was concluded. In instances where a mouse failed to locate the platform within the allotted time, it was guided to the platform and remained there for 30 s. A stationary camera positioned 1.5 m above the water surface recorded both the time taken to locate the platform and the swimming trajectory within the 60-s duration. 48 h post-training, the platform was removed from the maze to assess spatial memory. A digital device connected to a computer was employed to document the latency to the initial platform crossing, the frequency of crossings over the target platform area, and the duration spent in the target quadrant during the 60-s probe trial.

### Novel Object Recognition (NOR) test

An arena, measuring 50 cm × 50 cm × 50 cm, is utilized to house objects A and B in its corners. During the training phase of the test, each mouse is allotted 5 min to freely explore and acclimate to the environment. Following each familiarization session, the arena is sanitized using 70% ethanol. After a 24-h interval, one of the original objects is replaced with a novel object, C, which is distinguished by its unique shape and color. The mice are then given 5 min to explore the two objects. The object recognition time is assessed by a researcher who is blinded to the experimental conditions. Subsequently, the mice’s preferences for the novel object C and the familiar object A are quantified.

### Thioflavin-S staining

The brain sections underwent three washes with PBS. Following a 5 s incubation in 0.25% acetic acid, the sections were immersed in a 1% thioflavin-S (Thio-S) solution (Sigma-Aldrich) at 37 °C for 30 min in the dark. Subsequently, differentiation was achieved using 50% ethanol. Imaging was conducted using a Zeiss LSM880 confocal microscope, and fluorescence intensity quantitative analysis was performed with ImageJ software.

### Measurement of soluble and insoluble Aβ

Hippocampal samples were homogenized in 100 µL RIPA buffer (Beyotime, cat# P0013B), composed of 50 mM Tris (pH 7.4), 150 mM NaCl, 1% Triton X-100, 1% sodium deoxycholate, and 0.1% SDS. This was followed by centrifugation at 14,000 × g for 20 min. The resulting supernatants were collected for the quantification of soluble Aβ. To extract insoluble Aβ, the pellet fractions were further homogenized with 100 µL of SDS buffer, containing 10% SDS, 250 mM Tris, at pH 6.8. The concentration of soluble and insoluble Aβ_40_ and Aβ_42_ were analyzed with Human Aβ ELISA kits (R&D Systems, Cat# DAB142 for Aβ_42_, Cat# DAB140B for Aβ_40_) according to protocol.

### Viruses, reagents and antibodies

The adeno-associated virus (AAV) constructs, including AAV-GfaABC1D-shHDAC7-GFP and AAV-GfaABC1D-Scramble-GFP, AAV-GfaABC1D-HDAC7-3xFlag, AAV-GfaABC1D-GFP, AAV-GfaABC1D-3xFlag and AAV-GfaABC1D-Cre-NLS were constructed and packaged by Brain Case Technology (Shenzhen, China). The target sequence of HDAC7 shRNA is TGCGCTACAAACCCAAGAAAT. The AAV11 serotype was used in all experiments. TMP195 (a selective class IIa HDAC inhibitor, S8502) and IKK-16 (CHX, S72882) were procured from Selleck (Houston, TX, USA). The antibodies used in this study are listed in Supplementary Table S1.

### Western blotting

Mice were euthanized, and the hippocampi were immediately isolated on ice-cold PBS from the brains. The tissues were homogenized and subjected to centrifugation in RIPA buffer (Beyotime, cat# P0013B) supplemented with a mixture of protease inhibitors and phosphatase inhibitors. The protein concentration of the supernatant was determined using a BCA method (Pierce BCA protein assay kit, cat# 23225) and samples were denatured in SDS loading buffer. Proteins were then resolved via SDS-PAGE and transferred onto a nitrocellulose membrane (Whatman). The membrane was blocked with a buffer solution (Beyotime, cat # P0023B) and probed with primary antibodies at 4 °C overnight. Following incubation with an HRP-conjugated secondary antibody (Beyotime, cat# A0208/A0216/A0192), bands were visualized using the ECL detection system, and images were analyzed with ImageJ software. Densitometric analysis of the immunoblots was performed using ImageJ software, with β-actin serving as the loading control for normalization of target protein expression. Protein signals were detected using the ECL detection system and analyzed with ImageJ software (1.54p) (https://imagej.net/ij/index.html). The antibodies utilized for Western blotting analysis are listed in Supplementary Table S1.

### Real‐Time Quantitative (RT‐qPCR)

Real‐Time Quantitative Reverse Transcription‐PCR (qRT‐PCR) was performed as previously described. Total RNA was extracted using TRIzol reagent (Invitrogen, cat#15596018) and reverse transcription was conducted using the PrimeScript real time Master Mix (TAKARA, cat# RR047A). Prior to reverse transcription, genomic DNA was eliminated by incubating the reaction mixture at room temperature for 30 min. The total reaction volume for the RT-qPCR system was 20 μL, comprising 10 μL 2 × SYBR Green Master Mix (AGbio, AG11701), 2 μL forward primer, 2 μL reverse primer, 5 μL RNase/DNase-free H_2_O, and 1 μL pre-amplified cDNA (100 ng/μl). RT-qPCR was performed on a Bio-Rad Connect Real-Time PCR platform (Bio-Rad). Relative gene expression was determined using the 2^−ΔΔCT^ method, normalized to the housekeeping gene GAPDH. All primer used are listed in Supplementary Table S2.

### Preparation of Aβ

Synthetic human Aβ1–42 peptides (rPeptide) were initially dissolved in 1,1,1,3,3,3-Hexafluoro-2-propanol (HFIP) (Sigma-Aldrich) for 2 h to promote monomerization. Following this, the solvent was removed using a SpeedVac system (Thermo Fisher Scientific), and the peptides were subsequently stored at −80 °C. The Aβ_1–42_ monomers were then dissolved in dimethyl sulfoxide (DMSO) at a concentration of 1 mM and further diluted in F12 medium (Invitrogen) to achieve a final concentration of 100 μM. This solution was incubated at room temperature for 24 h. After incubation, the preparation underwent centrifugation at 14,000 × g for 20 min to sediment any fibrillar material, with the supernatant collected as the oligomer preparation.

### Cell culture and treatment

Primary mouse astrocytes were isolated from the cerebral cortices of postnatal C57BL/6 or *Hdac7*^flx/flx^ mice, following the protocol previously established [29]. Briefly, the cortices were dissected and mechanically dissociated, after which the resulting cell suspension was subjected to centrifugation and resuspended in a complete culture medium consisting of DMEM/F-12 supplemented with 10% fetal bovine serum (FBS) and 1% penicillin/streptomycin. The cells were then cultured in T75 flasks, with the medium being replaced every three days. After a period of 7–10 days, microglial contamination was eliminated by orbital shaking of the cultures at 220 rpm for 12 h. Following agitation, the suspended cells in the culture medium, including microglia, were extracted. The adherent cells remaining were subsequently cultured in Dulbecco’s modified Eagle’s medium (DMEM) F-12 supplemented with 10% FBS. Upon reaching confluence, the astrocytes were passaged to establish pure astrocytic cultures. The purity of the cells was verified using monoclonal anti-glial fibrillary acidic protein (GFAP) antibodies. For Aβ treatment, cells were incubated with Aβ (1 µM) serum‐free medium for 24 h. Control cells received an equivalent volume of vehicle (0.1% DMSO in serum-free medium) for the same duration. Then cells were collected for Western blotting, RT-qPCR or immunostaining analysis.

For the culture of primary cortical neurons, neurons were isolated from embryonic WT C57/B6 mice at embryonic days 17 to 19 as described previously [35]. The cortical tissues were excised and minced in ice-cold Hank’s buffered saline solution, followed by incubation in 0.25% trypsin at 37 °C for 15 min. The tissue was then gently triturated using a pipette for eight to ten iterations to dissociate the cells, resulting in a homogeneous cell suspension. The cell density was subsequently determined using a hemocytometer. Neurons were seeded in F-12 medium supplemented with 10% fetal bovine serum (FBS) on poly‐L‐lysine‐coated culture dishes at a density of 5–7 × 10^5^ cells per well. 4 h post-seeding, the initial medium was carefully removed, and 1.5 mL of fresh maintenance medium, consisting of 97% Neurobasal medium, 2% B27 supplement, and 1% Glutamax, pre-warmed to 37 °C, was added to each well. The maintenance medium was refreshed by replacing half of the volume every three days.

For collection of astrocyte-conditioned medium (CM), following Aβ treatment, the astrocytes were thoroughly washed with PBS for three times, and subsequently cultured with serum-free neuronal medium (97% Neurobasal medium, 2% B27 supplement, and 1% GlutaMAX) for 24 h. The CM were then collected and used to treat mouse cortical neurons. On day 9 of culture, the cortical neurons were exposed to equivalent volumes of astrocyte-CM for a period of 24 h. For non-conditioned medium (Non-CM) control, fresh neuronal medium (97% Neurobasal, 2% B27, 1% GlutaMAX) was incubated under the same culture conditions without any cells and then added to neurons. Neuronal viability was assessed using the Calcein AM/EthD-1 method, facilitated by the Viability/Cytotoxicity Assay Kit for Animal Live & Dead Cells (Proteintech, PF00008).

### Co-immunoprecipitation

For the coimmunoprecipitation experiments, primary cultured astrocytes were subjected to lysis using IP buffer (Beyotime, cat# P0013) supplemented with 1% protease inhibitors and phosphatase inhibitors, on ice for a duration of 10 min. Subsequently, the lysates were centrifuged at 12,000 × g for 15 min. The resulting soluble supernatants, containing 1 mg of total protein, were incubated with anti-NF-κB (Cell Signaling, 8242S, 1:100), anti-IKKα (Abcam, ab32041, 1:100), or anti-IKKβ (Abcam, ab32135, 1:100) antibodies under gentle rocking conditions at 4 °C overnight. This was followed by a 2-h incubation with protein A + G agarose. The agarose beads were then washed three times with PBS and resuspended in a sample buffer composed of 50 mM Tris·HCl, pH 7.6, 2% (wt/vol) SDS, 10% (vol/vol) glycerol, 10 mM DTT, and 0.2% (wt/vol) bromophenol blue, followed by boiling at 95 °C for 10 min. Finally, the samples were subjected to analysis via SDS-PAGE and immunoblotting.

### Immunofluorescence staining

For animal experimentation protocol, mice were anesthetized with isoflurane, followed by intracardial perfusion with saline and subsequently with a 4% paraformaldehyde (PFA) solution in 0.1 M phosphate buffer at pH 7.4. The brains were then extracted and further fixed in a 4% PFA solution for 12 h, after which they were subjected to cryoprotection in sequential 20% and 30% sucrose solutions. Brain sections with a thickness of 35 μm, were prepared using a cryostat microtome (CM1900, Leica, Germany). Initially, the brain sections were washed in PBS and blocked in a buffer containing 3% bovine serum albumin (BSA) and 0.5% Triton X-100 for 1 h. Subsequently, the sections were then incubated with indicated primary antibodies at 4℃ overnight. Following PBS washes, the sections were incubated with secondary antibodies conjugated to Alexa-Fluor 405/488/5555/647 at 37 °C for 1 h. For nuclear staining, the brain sections were incubated with DAPI (Beyotime, cat# C1002) for 10 min at room temperature under light-protected conditions. The sections were washed three times with PBS and mounted onto slides. Confocal images were acquired using a Zeiss LSM880 confocal microscope. Details information of antibodies used for immunofluorescence staining are listed in Supplementary Table S1.

For Fluoro Jade C staining, following incubation with the NeuN antibody, sections were incubated with secondary antibody conjugated to Alexa-Fluor 555 at 37℃ for 1 h. After washing with PBS for three times, brain sections were then subjected to Fluoro Jade C staining according to the manufacturer’s instructions. Images were taken using a using a Zeiss LSM880 confocal microscopy. The number of FJC spots was quantified using ImageJ software.

For the *in vitro* experiments, primary astrocytes were fixed with 4% paraformaldehyde for 15 min and subsequently permeabilized using a 0.5% Triton X-100 phosphate buffer for 30 min. This was followed by incubation in a phosphate buffer containing 0.1% Triton X-100 and 3% bovine serum albumin (BSA) to minimize nonspecific binding. The cells were then incubated with the specified primary antibodies at 4 °C overnight. Following incubation, the cells were washed three times with PBS, with each wash lasting 5 min. Subsequently, they were incubated with fluorophore-conjugated secondary antibodies for 1 h at 37 °C in a dark environment. Nuclear staining was performed using DAPI (Beyotime, cat# C1002) for 5 min. Detailed information regarding the antibodies used for immunofluorescence staining is provided in Supplementary Table S1.

### Immunohistochemistry staining

The brain sections were washes with PBS containing 0.2% Tween 20 (PBST) for three intervals of 5 min each. Subsequently, sections were incubated with 3% hydrogen peroxide in absolute ethanol for 15 min, followed by treatment with 0.5% Triton X-100 for 30 min. Nonspecific binding sites were then blocked using 3% bovine serum albumin (BSA) for 30 min at room temperature. The sections were incubated with anti-amyloid 6E10 (Biolegend, cat# 803001) with a dilution of 1:500 at 4 °C overnight. Post-incubation, the sections were washed again with PBST for three intervals of 5 min each. The immunoreaction was developed using the HistostainTM-SP kit and visualized with diaminobenzidine, resulting in a brown coloration. The sections were subsequently dehydrated using a graded ethanol series and mounted with coverslips. Microscopic images were captured using an Olympus BX60 microscope (Tokyo, Japan).

### Image quantification

Images were captured using a Zeiss LSM880 confocal microscope, maintaining consistent acquisition parameters across samples within each experimental set. For each mouse, 3–4 brain sections encompassing the hippocampus were analyzed per stain. In APP/PS1 mice, 4–5 Aβ plaques per section were randomly selected for imaging, and the mean fluorescence intensity of HDAC7, C3, and GFAP was measured within the image field centered on the plaque. In WT mice, which do not develop plaques, images were acquired from the same hippocampal subregions at randomly selected positions, with the same number of image fields per mouse as for APP/PS1 mice. For measurements in the CA1 region, the CA1 pyramidal cell layer was manually outlined as a region of interest (ROI). The area (mm^2^) of the ROI was measured, and the mean fluorescence intensity of cleaved Caspase3 was measured within the ROI. The number of NeuN⁺ cells within the same ROI was counted, and cell density was calculated as cells/mm^2^. For correlation analysis, 10 astrocytes located directly adjacent to the plaque border were randomly selected per mouse, and the mean fluorescence intensity of HDAC7 and C3 within GFAP⁺ astrocytes was measured (total 60 cells from 6 mice). For Fluoro-Jade C staining, FJC-positive puncta within the dentate gyrus were quantified utilizing the “Analyze Particles” function in Image J. The nuclear-to-cytoplasmic ratio for NF-κB translocation was determined on a per-cell basis. All image quantifications were conducted by an investigator blinded to both genotype and treatment conditions.

### Golgi staining and sholl analysis

Golgi staining in brain tissue from 8‐month‐old mice was performed using a FD Rapid Golgi Stain Kit (FD NeuroTech, PK401) following the manufacturer’s protocol. Mice were sacrificed post-anesthetization with isoflurane and brains were embedded in A + B solution and incubated in the dark for 14 days. Subsequently, the brains were transferred to C solution and incubated in the dark for 10 days. Brain tissue was then cut into 100‐µm sections using a Vibratome (VT1200S; Leica, Germany) and stained with a D + E solution. Images were acquired using a Nikon Ni-E microscope (Nikon, Japan), employing a 100 × oil immersion lens for the visualization of dendritic spines in CA1 neurons or 10 × lens for the visualization of dendritic complexity.

Neurons that showed intact and complete dendritic arbors, and relative isolation to other neurons were selected for Sholl analysis. The dendritic spines were imaged from apical dendritic branches at least 200 μm away from the cell soma. Analysis was conducted with ImageJ using the Simple Neurite Tracer plugin following previously described methods [36].

### Bulk RNA-seq and pathway analysis

Mice were anesthetized using isoflurane, and the hippocampi were promptly isolated in ice-cold PBS from the brains. Total RNA was extracted employing the TRIzol reagent (Invitrogen, cat# 15596018). RNA-seq library construction and sequencing were conducted by Shenzhen Climb Technology. For the bulk RNA-seq analysis, to get high quality clean reads, reads were further filtered by FASEP (version 0.23.4). The high-quality reads were aligned to the mouse genome (GRCm38/mm10) using HISAT (version 2. 2.2.1). The mapped reads of each sample were assembled by using StringTie (version 2.2.1). Differentially expressed genes (DEGs) between HDAC7-overexpressing and control GFP groups were identified with the DESeq2 R package, based on the criteria of |log2 (fold change) |> 0 and P-value < 0.05. Pathway enrichment analysis of these DEGs, including Gene Ontology and KEGG pathway analyses, was conducted using Metascape (https://metascape.org) [37]. The RNA-seq raw data have been deposited in the NCBI Sequence Read Archive (SRA) under BioProject accession PRJNA1471440.

### Statistical analysis

Statistical analyses were performed with GraphPad Prism 9.5 (LaJolla, CA, USA). Unpaired Student’s t-test was used to determine differences between two groups. One-way or two-way analysis of variance (ANOVA) followed by post hoc comparisons was used to determine differences among three or more groups as indicated in the figure legends. The statistically significance levels were set at p < 0.05 (*), p < 0.01 (**), p < 0.001 (***) with a confidence interval of 95%. Data were expressed as mean ± SEM. All samples or animals were included for statistical analysis unless otherwise noted in pre-established criteria. Post-hoc power calculations were performed using G*Power 3.1 (α = 0.05, two-tailed where applicable). The results, including effect sizes (Cohen’s d for t-tests, η^2^/partial η^2^ for ANOVA, and r for Pearson correlation) and power (1-β) for all key experiments, are summarized in Supplementary Table S8.

## Results

### HDAC7 is uniquely upregulated in plaque-associated neurotoxic astrocytes in APP/PS1 mice

**Fig. 1:**
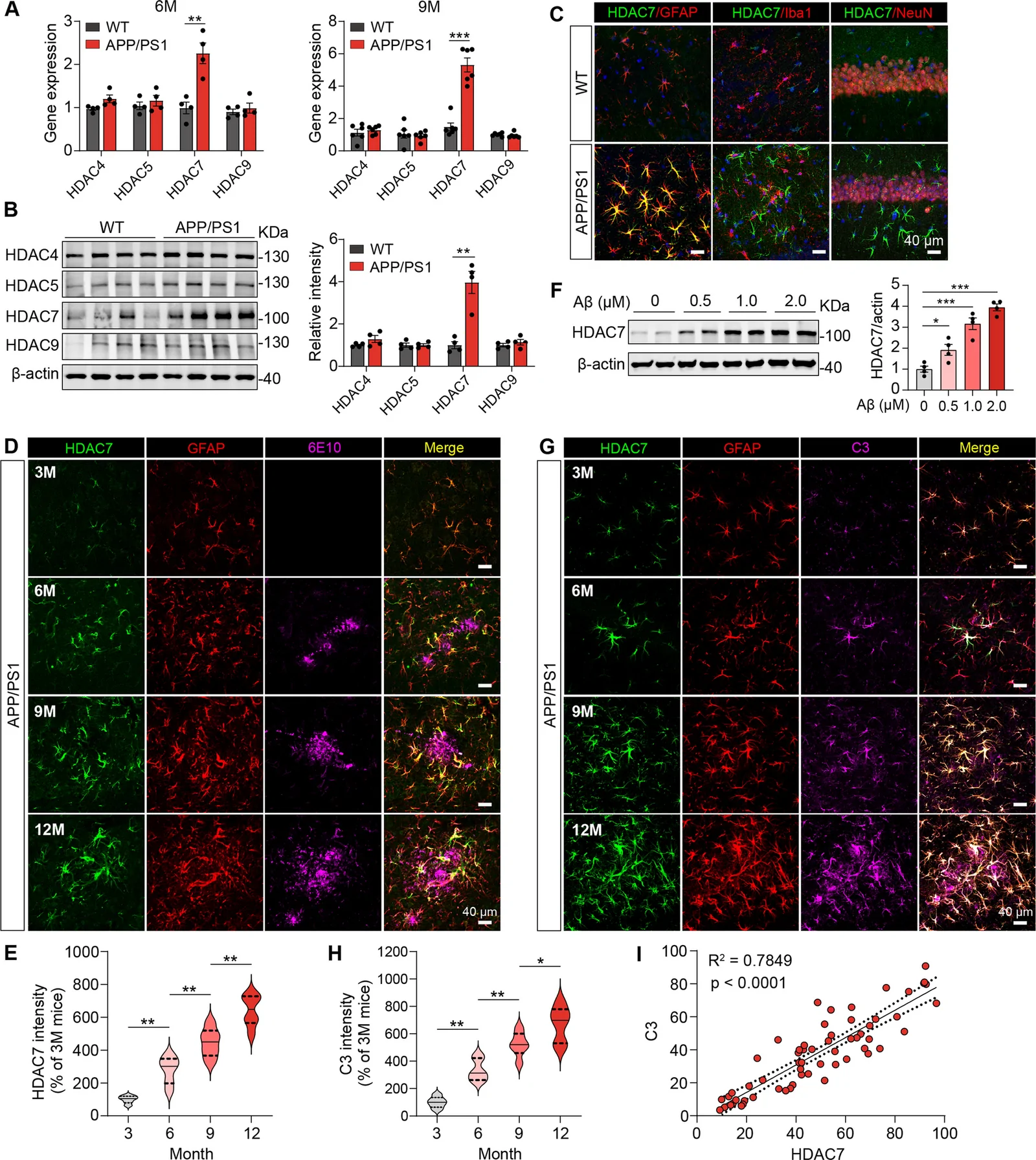
HDAC7 level is upregulated in plaque-adjacent neurotoxic astrocytes in a mouse model of Aβ amyloidosis. **A** RT-qPCR analysis of class IIa HDACs (HDAC4, 5, 7 and 9) mRNA level in the hippocampal samples of 6- and 9-month-old WT and APP/PS1 mice. n = 4–6 mice per group. **B** Western blotting analysis and quantification of HDAC4, 5, 7 and 9 protein levels in the hippocampal lysates of 9-month-old WT and APP/PS1 mice. n = 4 mice per group. **C** Representative immunostaining images of HDAC7 and glial fibrillary acidic protein (GFAP)/ionized calcium binding adapter molecule 1 (Iba1)/neuronal nuclei (NeuN) in the brain of 9-month-old WT and APP/PS1 mice. **D** Representative immunostaining images of HDAC7, GFAP and Aβ (6E10) in the hippocampus of 3-, 6-, 9- and 12-month-old APP/PS1 mice. **E** Quantification of HDAC7 intensity in GFAP.^+^ cells in the hippocampus of APP/PS1 mice. n = 6 mice per group. **F** Western blotting analysis and quantification of HDAC7 level in primary cultured mouse astrocytes treated with Aβ oligomer or vehicle. n = 4 per group. **G** Representative immunostaining images of HDAC7, GFAP and complement C3 in the hippocampus of 3, 6, 9 and 12-month-old APP/PS1 mice. **H** Quantifications of C3 intensity in GFAP-positive cells in the hippocampus of APP/PS1 mice. n = 6 mice per group. **I** Correlation analysis of HDAC7 and C3 expression levels in plaque-adjacent astrocytes in the brains of APP/PS1 mice, n = 60 cells from 6 mice. Statistical significance was determined by unpaired Student’s t test (A, B) or one-way ANOVA with Tukey’s post hoc analysis (E, F and H). Data are shown as mean ± SEM, *p < 0.05, **p < 0.01, ***p < 0.001

Our previous study has demonstrated that class IIa HDACs are integral to the regulation of astrocyte function during tau pathology, a characteristic hallmark of AD [29]. To investigate the potential involvement of class IIa HDACs in Aβ pathology, we firstly analyzed their transcript levels in the APP/PS1 transgenic mouse model. RT-qPCR analysis indicated that HDAC7, but not HDAC4, HDAC5, or HDAC9, was the sole one that exhibits increase in the hippocampus of 6- and 9-month-old APP/PS1 transgenic mice (Fig. 1A). This mRNA expression pattern is further validated at protein level, whereby a unique elevation of HDAC7 among class IIa HDAC family was observed in the hippocampus of APP/PS1 mice compared to wild-type (WT) mice (Fig. 1B). To elucidate the cellular distribution of HDAC7 alterations within the brain, we employed immunofluorescence co-staining with antibodies specifically targeting markers for neurons (NeuN), astrocytes (GFAP), and microglia (Iba1). The immunostaining results revealed that the upregulated HDAC7 is predominantly localized within astrocytes, rather than neurons or microglia, in the brain of APP/PS1 mice (Fig. 1C). Furthermore, HDAC7 expression in APP/PS1 brains was found to be highly enriched in astrocytes that were adjacent to Aβ plaques, and this phenomenon increased with disease progression (Fig. 1D and E). Given that Aβ burden escalates with age in the APP/PS1 transgenic model, we explored the potential contribution of Aβ to the upregulation of HDAC7 in astrocytes. In primary cultured astrocytes, treatment with Aβ oligomers resulted in a concentration-dependent increase in HDAC7 protein levels (Fig. 1F), indicating that Aβ may act as an upstream factor of HDAC7 elevation in astrocytes. Collectively, these findings imply that HDAC7 is increased in astrocytes in the context of Aβ pathology.

In the initial stage of AD, astrocytes are anticipated to attempt to maintain brain homeostasis by migrating towards Aβ plaques and facilitating Aβ clearance [38]. However, the persistent accumulation of Aβ, along with the consequent activation of microglia, induces astrocytes to transition into a neurotoxic reactive state, thereby promoting neuronal death and neurodegeneration [15, 39]. In light of these observations, we investigated the potential relationship between HDAC7 expression and astrocyte neurotoxicity. Through co-immunostaining with complement C3, HDAC7, and GFAP, we observed that the expression level of C3, a typical marker of neurotoxic reactive astrocytes, increased with disease progression in APP/PS1 mice (Fig. 1G and H), consistent with previous findings [15]. Importantly, the intensity of C3 signal in astrocytes was positively correlated with the expression level of HDAC7 (Fig. 1G and I). Collectively, these data indicate that HDAC7 is upregulated in astrocytes and is associated with the formation of neurotoxic astrocytes in APP/PS1 mice.

### Astrocyte-specific overexpression of HDAC7 induces the formation of neurotoxic reactive astrocytes and exacerbates cognitive deficits in mice

**Fig. 2:**
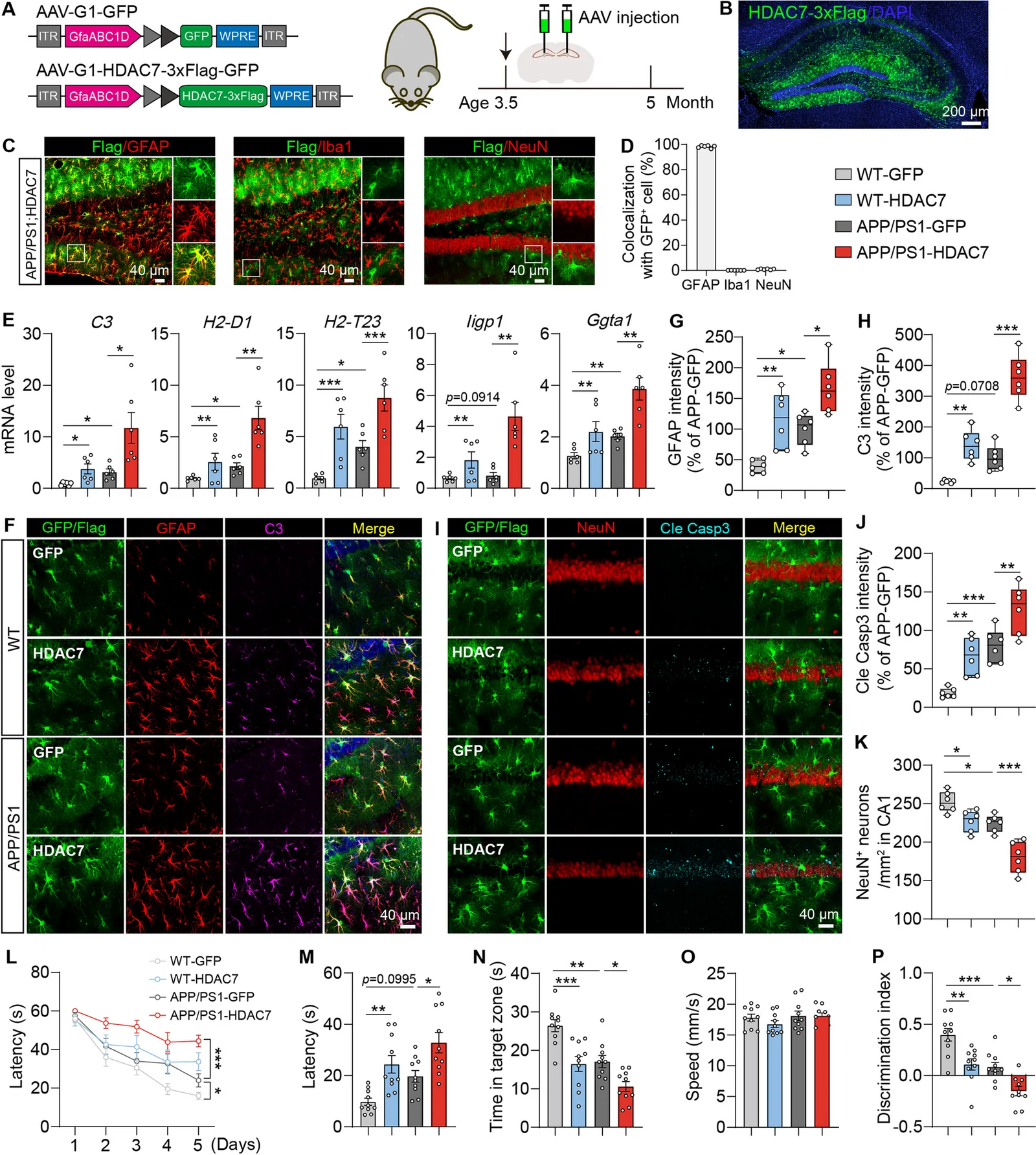
Overexpression of HDAC7 in astrocytes promotes the formation of neurotoxic reactive astrocytes and induces cognitive deficits in mice. **A** Schematic illustration of AAVs constructed with HDAC7 or control GFP and the in vivo experimental timeline. **B** Representative immunofluorescence image with anti-Flag antibody showing the expression of AAV-G1-HDAC7-3xFlag in the hippocampus of APP/PS1 mice. **C** Representative immunostaining images of anti-Flag and cellular markers against astrocyte (GFAP), microglia (Iba1), or neuron (NeuN) in the hippocampus of APP/PS1 mice injected with AAV-G1-HDAC7-3xFlag. **D** The ratio of GFAP^+^ GFP^+^, Iba1^+^ GFP^+^ and NeuN^+^ GFP^+^ double positive cells in mouse brain sections. n = 6 mice per group. Scale bar: 40 μm. **E** RT-qPCR analysis of neurotoxic reactive astrocyte marker genes in the hippocampal tissue samples of WT and APP/PS1 mice with astrocytic HDAC7 overexpression. n = 6 mice per group. **F** Representative immunofluorescence images showing the expression of GFP, pan-reactive astrocyte marker GFAP, and neurotoxic astrocyte marker C3 in the hippocampal region of WT and APP/PS1 mice following HDAC7 overexpression. **G**,** H** Quantification of the intensity of GFAP and C3 in GFP^+^ cells, n = 6 mice per group. **I** Representative immunofluorescence images showing the expression of GFP, neuronal marker NeuN, and apoptotic marker cleaved Caspase3 in the hippocampal CA1 region. **J**, **K** Quantification of the intensity of cleaved Caspase3 and the number of NeuN^+^ cells in the CA1 area, n = 6 mice per group. **L**-**O** Morris water maze (MWM) behavioral analysis. **L** Escape latency during the training phase, **M** first latency to reach the platform, **N** exploring time in target zone, and **O** swimming speed during the probe test. **P** Discrimination index of mice in the Novel object recognition (NOR) behavioral test. For behavioral tests, n = 10 mice per group. Statistical significance was determined by one-way ANOVA (**E**, **G**, **H**, **J**, **K**, and **M**-**P**) or two-way ANOVA (L) with Tukey’s post hoc analysis. Data are shown as mean ± SEM, *p < 0.05, **p < 0.01, ***p < 0.001, n. s, not significant

To explore the role of HDAC7 upregulation in astrocytes under both physiological and pathophysiological conditions, we conducted astrocyte-specific overexpression of HDAC7 in the hippocampus of 3.5-month-old WT and APP/PS1 mice (Fig. 2A). Astrocyte-specific gene delivery was achieved through an adeno-associated virus (AAV) vector mediated viral strategy under the astrocyte-specific GfaABC1D (G1) promoter [29, 40]. 1.5-month post injection with AAV-G1-HDAC7-3xFlag or AAV-G1-GFP, the expression of the astrocyte-targeted AAV in the hippocampus was verified via confocal microscopy (Fig. 2B). Immunofluorescence co-staining revealed that the HDAC7-3xFlag was specifically expressed in astrocytes (GFAP), rather than microglia (Iba1) or neurons (NeuN) (Fig. 2C and D).

Analysis of genes encoding neurotoxic reactive astrocyte markers, specifically *C3*, *H2-T23*, *H2-D1*, *Ggta1*, and *Iigp1,* revealed that HDAC7 overexpression significantly increased the expression of these genes in the hippocampus of WT mice, and further intensified this upregulation in APP/PS1 mice (Fig. 2E). Immunostaining analysis corroborated these gene level findings, showing that the expression levels of C3 and GFAP, indicative of neurotoxic reactive astrocytes, were significantly increased by HDAC7 overexpression in WT mice, and was further augmented in HDAC7-overexpressing APP/PS1 mice (Fig. 2F-H). Furthermore, the impact of astrocytic HDAC7 on hippocampal neurons was evaluated through co-immunostaining with neuronal marker NeuN and cleaved Caspase3. Compared with GFP controls, a significant increase in apoptosis (cleaved Caspase3^+^) and neuronal loss (NeuN^+^) was observed in pyramidal neurons of mice with astrocytic HDAC7 overexpression (Fig. 2I-K).

The cognitive function of mice with astrocytic HDAC7 overexpression was measured using behavioral tests. Through Morris Water Maze (MWM) test, we found that HDAC7 overexpression led to impairments in spatial learning and memory in WT mice and exacerbated these behavioral deficits in APP/PS1 mice (Fig. 2L-N). This was evidenced by the increased latency to reach the platform during the training phase and the probe phase (Fig. 2L and M), and reduced platform zone exploring time in the probe phase (Fig. 2N), with the swimming speed remained comparable across all experimental groups (Fig. 2O). The Novel Object Recognition (NOR) test also indicated that mice with astrocytic HDAC7 overexpression exhibited reduced discrimination for novel object, implying impaired cognitive function (Fig. 2P). Additionally, we investigated whether upregulation of HDAC7 in astrocytes has an impact on the progression of Aβ pathology. Interestingly, immunohistochemistry staining with anti-6E10 antibody showed that HDAC7 overexpression in astrocytes accelerated the formation of Aβ plaques in the hippocampus of APP/PS1 mice (Supplementary Figure S1A and B). Moreover, ELISA analysis indicated that the levels of both soluble and insoluble Aβ_40_ and Aβ_42_ species, were significantly increased in the hippocampal extracts of APP/PS1 mice with HDAC7 overexpression (Supplementary Figure S1C).

Taken together, these data indicates that HDAC7 overexpression promotes the neurotoxic phenotypic alteration of astrocytes, leading to neuronal loss, cognitive dysfunction, and exacerbated Aβ pathology in mice.

### HDAC7 regulates genes involved in neuroinflammation and neuronal function, and activates NF-κB signaling* in vivo*

**Fig. 3:**
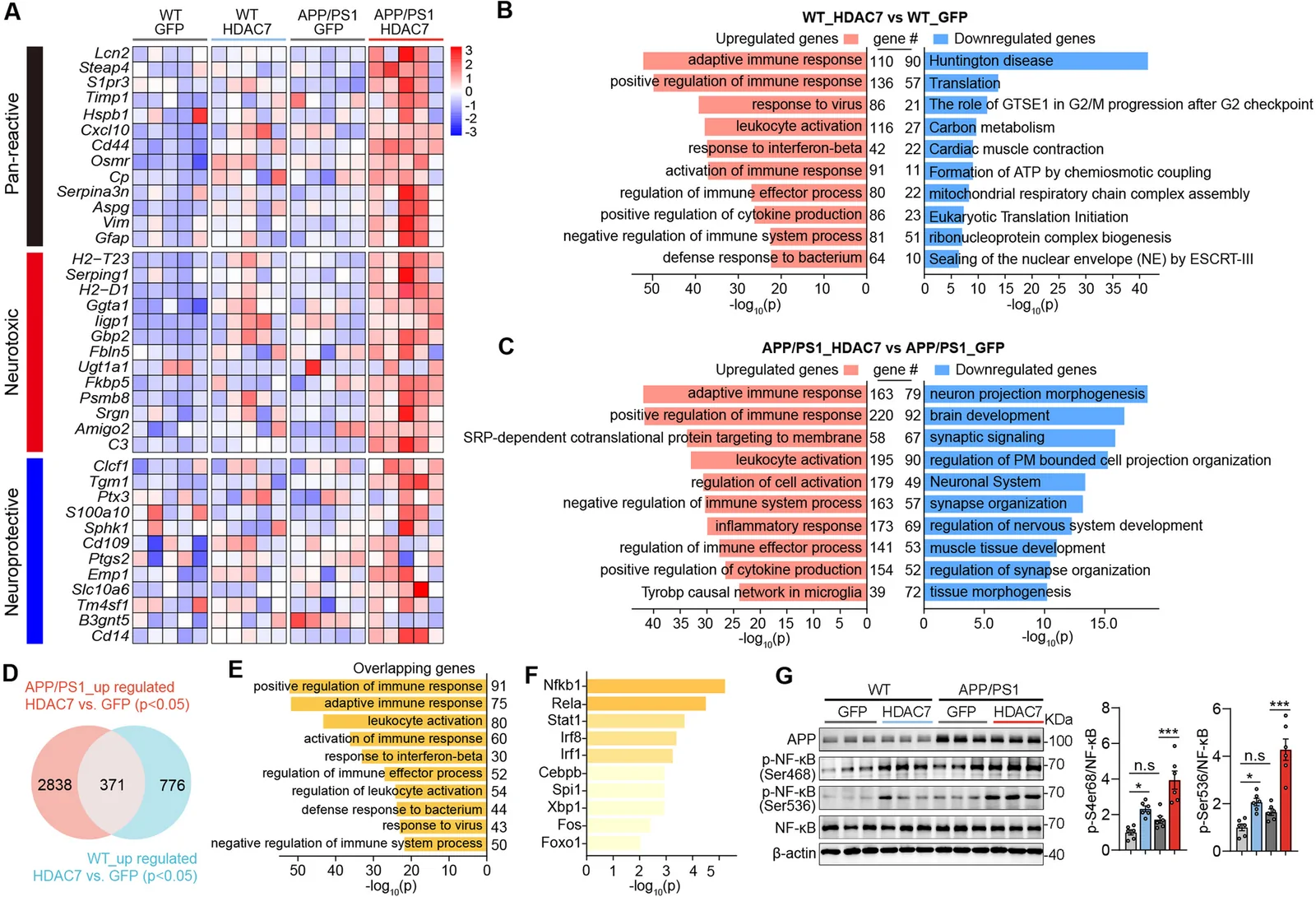
HDAC7 regulates genes involved in astrocyte reactivity and neuronal function involving NF-κB signaling activation. **A** Heatmap depicting the expression patterns of pan-reactive astrocyte markers, neurotoxic reactive astrocyte markers and neuroprotective astrocyte markers in the hippocampal tissues of WT and APP/PS1 mice with astrocytic HDAC7 overexpression. **B** Bar graph showing the top 10 enriched functional terms for up-regulated and down-regulated DEGs due to HDAC7 overexpression in WT mice. **C** Bar graph showing the top 10 enriched functional terms for up-regulated and down-regulated DEGs due to HDAC7 overexpression in APP/PS1 mice. **D** A Venn diagram showing the overlap of upregulated DEGs identified by astrocytic HDAC7 overexpression in WT and in APP/PS1 mice. **E** Enriched functional terms of the 371 overlapped DEGs between WT and APP/PS1 group, colors indicating p-values. **F** Heatmap showing the potential transcriptional factors that regulating the 371 overlapped DEGs according to TRRUST database. **G** Western blotting analysis and quantification of NF-κB, p-NF-κB (Ser536), and total APP in the hippocampal tissue samples of WT and APP/PS1 mice. n = 6 mice per group. Statistical significance was determined by one-way ANOVA with Tukey’s post hoc analysis. Data are shown as mean ± SEM, *p < 0.05, **p < 0.01, ***p < 0.001, n. s, not significant

To investigate the transcriptional consequences of astrocyte-specific HDAC7 overexpression, we conducted bulk RNA sequencing (RNA-seq) on hippocampal tissues of APP/PS1 and WT mice infected with AAV-G1-HDAC7-3xFlag or control AAV-G1-GFP. We subsequently analyzed the expression patterns of previously established pan-reactive and neurotoxic astrocyte substate markers [6]. Neuroprotective substate markers were also analyzed. Interestingly, pan-reactive marker genes such as *Cxcl10*, *Cd44* and *Aspg*, and neurotoxic reactive astrocyte marker genes including *C3*, *H2-D1*, *H2-T23*, *Ggta1*, *Gbp2* and *Psmb8,* exhibited significant upregulation in both WT and APP/PS1 mice following astrocyte HDAC7 overexpression (Fig. 3A and supplementary Figure S2). A modest upregulation of certain neuroprotective astrocyte markers was also observed (Fig. 3A and Supplementary Figure S2), aligning with the mixed reactive astrocyte signatures commonly reported in disease models [6, 41].

Among the 3003 differentially expressed genes (DEGs) identified between the HDAC7 and GFP group in WT mice, 1147 DEGs were upregulated and 1856 DEGs were downregulated (Supplementary Table S3). Using online Metascape, functional enrichment analysis demonstrated that the upregulated genes in WT group are predominantly associated with immune response pathways, including the adaptive immune response, response to interferon-beta, and positive regulation of cytokine production (Fig. 3B and Supplementary Table S4). In contrast, the downregulated pathways are primarily linked to Huntington disease, translation and Carbon metabolism (Fig. 3B and Supplementary Table S4). In APP/PS1 mice, HDAC7 elicits a more profound alteration in global transcriptomics, with 3209 genes upregulated and 2226 genes downregulated (Supplementary Table S5). Consistent with the transcript profile observed in WT mice, the upregulated genes in APP/PS1 group are enriched in immune and inflammatory related pathways, such as adaptive immune response, regulation of cell activation and inflammatory response (Fig. 3C and Supplementary Table S6). Conversely, the downregulated genes are enriched in neuronal and synaptic pathways, including neuron projection morphogenesis, brain development, synaptic signaling, and synapse organization (Fig. 3C and Supplementary Table S6).

The similarity in enriched pathways among upregulated DEGs in WT and APP/PS1 suggests that these genes may be involved in the neurotoxic effects observed following astrocytic HDAC7 overexpression. To identify a core set of HDAC7-driven transcriptional changes that occur independently of Aβ pathology, we intersected the upregulated DEGs in WT and APP/PS1 mice during HDAC7 overexpression. This approach identifies for common downstream effectors of HDAC7 in astrocytes excluding AD-specific modifiers. We identified a cohort of 371 overlapping DEGs (Fig. 3D and Supplementary Table S7). Gene Ontology GO and pathway analyses of these overlapping DEGs revealed significant enrichment in immune processes and inflammatory response pathways (Fig. 3E). Using the TRRUST database, we predicted the top 10 potential transcription factors (TFs) regulating these overlapping DEGs, including Nfkb1, Rela, and Stat1 (Fig. 3F). NF-κB is recognized as a key regulator of inflammation in the brain, with its activation promoting the expression of neurotoxic reactive astrocyte marker genes such as *C3*, *HT-D1* and *Gbp2* [15, 39]. Given the central role of NF-κB in neuroinflammation and astrocyte reactivity, as well as its enrichment as a TF for HDAC7-driven DEGs, we postulated that NF-κB might function as a downstream effector of HDAC7. Consistent with this hypothesis, western blotting analysis of hippocampal tissues revealed that HDAC7 overexpression in WT mice significantly increased the phosphorylation levels of NF-κB at Ser468 and Ser536, with further exacerbation observed in APP/PS1 mice (Fig. 3G), thereby indicating activation of NF-κB signaling. The concurrent activation of NF-κB signaling and the induction of a neurotoxic gene signature in the brains of HDAC7-overexpressing mice suggests that NF-κB signaling may function as a downstream effector of neuronal damage.

### *HDAC7 regulates Aβ-induced NF-κB signaling activation *via* deacetylating IKKα and IKKβ*

**Fig. 4:**
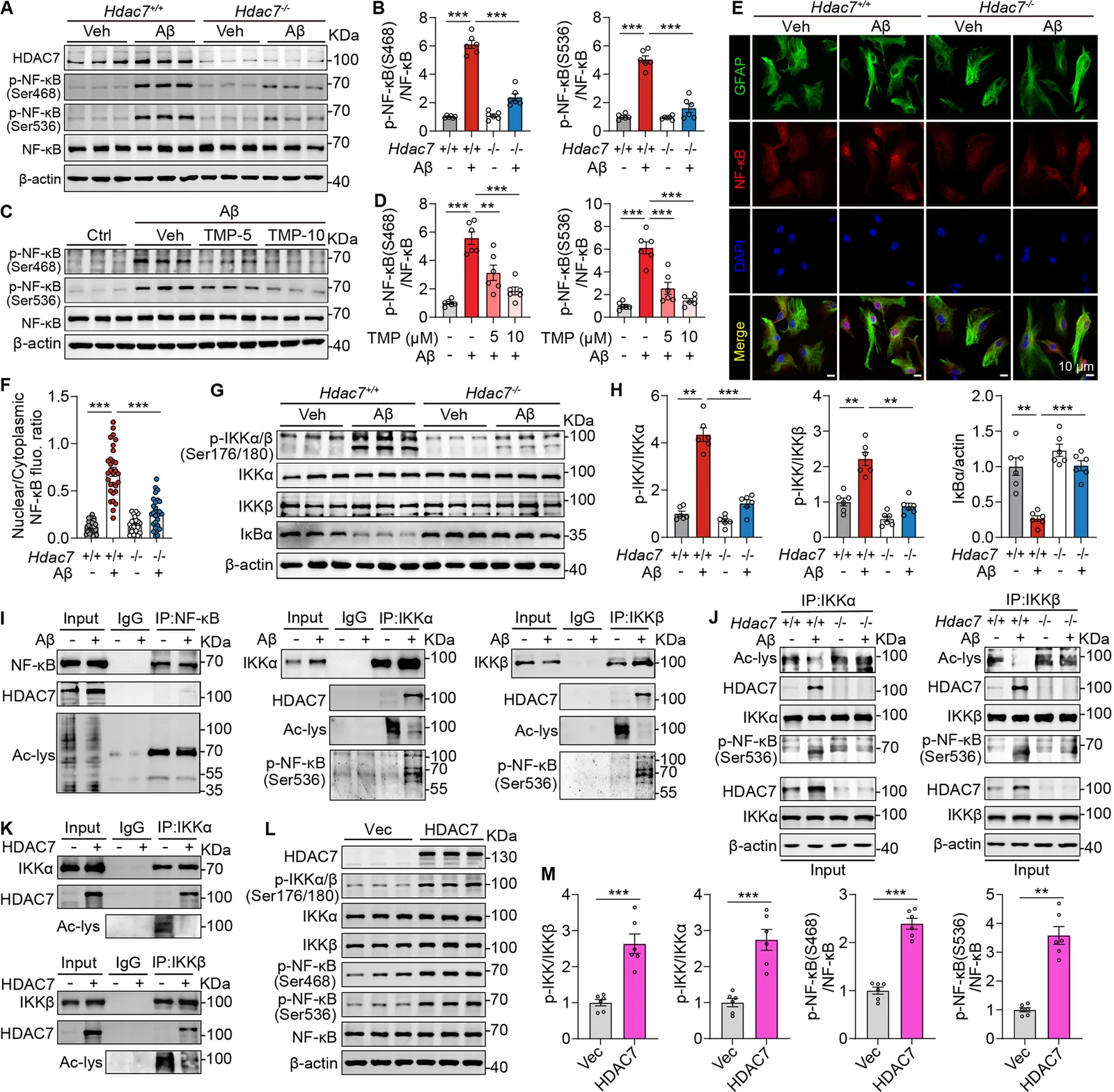
HDAC7 regulates Aβ-induced NF-κB signaling activation via deacetylating IKKα and IKKβ.** A**,** B** Western blotting analysis and quantification of HDAC7, p-NF-κB (Ser468), p-NF-κB (Ser536), and NF-κB in primary cultured control and HDAC7 knockout astrocytes treated with Aβ oligomer (1 μM). n = 6 per group. **C**,** D** Western blotting analysis and quantification of p-NF-κB (Ser468), p-NF-κB (Ser536), and NF-κB in primary astrocytes treated with Aβ oligomer and various concentration of TMP195 for 24 h. n = 6 per group. **E** Representative immunostaining images of NF-κB in control and HDAC7 null astrocytes treated with Aβ oligomer or vehicle for 24 h. Scale bar:10 μm. **F** Quantification of nuclear/cytoplasmic NF-κB ratio in E. n = 27–30 cells per group. **G**,** H** Western blotting analysis and quantification of p-IKKα/β (Ser176/180), IKKα, IKKβ, and IκBα in control and HDAC7 null astrocytes treated with Aβ oligomer. n = 6 per group. **I** Co-immunoprecipitation (Co-IP) assay showing that HDAC7with anti-NF-κB, anti-IKKα, and anti-IKKβ antibodies in control and Aβ-stimulated primary astrocytes. Representative blots showing the interaction between NF-κB, IKKα, and IKKβ with HDAC7, their acetylation levels, and the levels of IKKα- and IKKβ-bound p-NF-κB (Ser536). **J** Co-IP assay showing the acetylation levels of IKKα and IKKβ and the levels of IKKα- and IKKβ-bound p-NF-κB (Ser536) in control and HDAC7 null astrocytes treated with Aβ. **K** Co-IP assay demonstrating the interaction between IKKα, and IKKβ with HDAC7, and their acetylation levels in HDAC7-overexpressing astrocytes. **L**,** M** Western blotting analysis and quantification of p-IKKα/β (Ser176/180), IKKα, IKKβ, p-NF-κB (Ser468), p-NF-κB (Ser536), and NF-κB in control and HDAC7-overexpressing astrocytes. n = 6 per group. Statistical significance was determined by one-way ANOVA with Tukey’s post hoc analysis (**B**, **D, F** and **H**) or unpaired Student’s t test (**M**). Data are shown as mean ± SEM, *p < 0.05, **p < 0.01, ***p < 0.001, n. s, not significant

Aβ oligomers can readily elicit NF-κB activation in cultured astrocytes, thereby promoting the neurotoxicity of astrocytes [39]. To investigate the potential involvement of HDAC7 in this process, we assessed NF-κB signaling activity in WT and HDAC7-knockout astrocytes following Aβ oligomer stimulation. Primary astrocytes from *Hdac7*^flx/flx^ mice were transduced with either AAV-GfaABC1D-Cre-NLS to achieve HDAC7 deletion or a control AAV vector, and subsequently treated with 1 μM Aβ oligomer. As expected, the deletion of HDAC7 significantly reduced Aβ-induced phosphorylation of NF-κB p65 at Ser468 and Ser536 (Fig. 4A and B). Similar inhibitory effects on Aβ-induced NF-κB phosphorylation were observed with pharmacological inhibition of HDAC7 using TMP195 in cultured astrocytes (Fig. 4C and D). Further immunostaining analysis of NF-κB corroborated the attenuating effects of HDAC7 deletion on Aβ-induced NF-κB nuclear translocation (Fig. 4E and F). Consequently, we examined the activity of upstream kinase IKK in this process. Western blotting analysis revealed that HDAC7 deletion significantly decreased Aβ-induced upregulation of IKKα and IKKβ phosphorylation levels at Ser176 or Ser180 (Fig. 4G and H). In addition, HDAC7 deletion cells showed increased IκBα protein level compared to WT cells upon Aβ treatment (Fig. 4G and H). Collectively, these findings suggest a critical involvement of HDAC7 in modulating NF-κB signaling in response to Aβ oligomers.

To gain mechanistic insights into HDAC7 and IKK-NF-κB signaling activation, we conducted co-immunoprecipitation (Co-IP) assays with anti-NF-κB, anti-IKKα, anti-IKKβ antibodies in astrocytes stimulated with Aβ. Notably, following Aβ treatment, HDAC7 was observed to associate with IKKα and IKKβ, but not with NF-κB (Fig. 4I). Furthermore, Aβ stimulation resulted in a pronounced reduction in the acetylation levels of IKKα and IKKβ, whereas no such reduction was observed on NF-κB (Fig. 4I), indicating that HDAC7 might activates NF-κB signaling through deacetylating IKKα and IKKβ rather than direct interaction with NF-κB. The deacetylated form of IKKα and IKKβ showed increased kinase activity, as validated by the phosphorylation level of NF-κB at Ser536 via the immunoprecipitation-based kinase activity assay (Fig. 4I). Collectively, these findings suggest that the deacetylation of IKKα/β by HDAC7 plays a role in its activation upon Aβ stimulation.

To further substantiate the involvement of HDAC7 in the deacetylation and subsequent activation of IKKα/β, we examined the acetylation status of IKKα and IKKβ in HDAC7 knockout astrocytes. Following Aβ treatment, Co-IP assay demonstrated that the deletion of HDAC7 effectively reinstated the acetylation levels of both IKKα and IKKβ, which was associated with a reduction in IKK kinase activity, as evidenced by decreased phosphorylation of NF-κB at Ser536 (Fig. 4J). These data highlight the pivotal role of HDAC7 in the deacetylation and activation of IKKα/β during Aβ stimulation. We then asked whether upregulation of HDAC7 alone could induce IKKα/β deacetylation and downstream NF-κB signaling activation in the absence of Aβ. In primary astrocytes overexpressing HDAC7, we observed that HDAC7 directly interacted with IKKα and IKKβ, leading to reduced acetylation levels of both proteins (Fig. 4K), mirroring the effects observed following Aβ treatment. Furthermore, western blotting analysis revealed that HDAC7 overexpression activated the IKK-NF-κB signaling pathway in primary astrocytes (Fig. 4L and M). Collectively, these findings identify IKK deacetylation as a key downstream effector that links HDAC7 to Aβ-induced NF-κB activation in astrocytes.

### HDAC7 drives the neurotoxicity of Aβ-challenged astrocytes via IKK-NF-κB signaling pathway

**Fig. 5:**
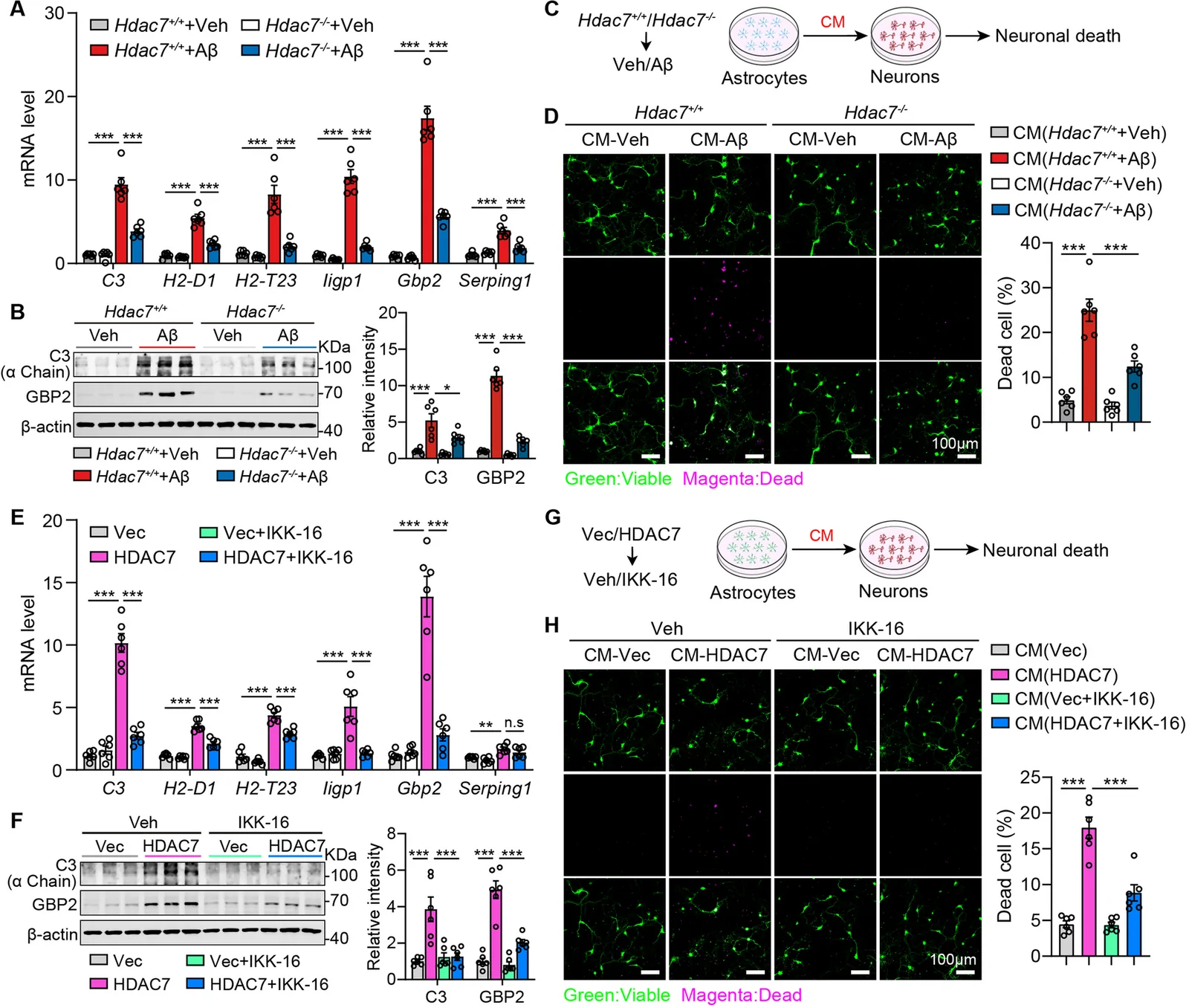
HDAC7 regulates the neurotoxicity of astrocytes via the IKK-NF-κB signaling pathway. **A** RT-qPCR analysis showing the mRNA levels of neurotoxic astrocyte marker genes in primary cultured WT and HDAC7 null astrocytes treated with 1 μM Aβ or vehicle. n = 6 per group. **B** Western blotting analysis of C3 and GBP2 in Aβ-treated WT and HDAC7 null astrocytes. n = 6 per group. **C** Experimental scheme to test the effects of Aβ-stimulated astrocytes conditioned medium (CM) on neurons. **D** Calcein AM/EthD-1 staining showing cell viability (green: live cells) and death (megenta: dead cells) of primary neurons treated with CM from Aβ-treated WT and HDAC7 null astrocytes. n = 6 dishes per group. **E** RT-qPCR analysis of neurotoxic astrocyte marker genes in control and HDAC7-overexpressing astrocytes treated with IKK inhibition IKK-16 (5 μM) or vehicle for 6 h. n = 6 per group. **F** Western blotting analysis of C3 and GBP2 in control and HDAC7-overexpressing astrocytes treated with IKK-16 or vehicle. n = 6 per group. **G** Experimental scheme to examine the effects of HDAC7-overexpressing astrocytes CM on neurons. **H** Calcein AM/EthD-1 staining showing cell viability and death of primary neurons treated with CM from HDAC7-overexpressing astrocytes treated with IKK-16 or vehicle. n = 6 dishes per group. Statistical significance was determined by one-way ANOVA with Tukey’s post hoc analysis. Data are shown as mean ± SEM, *p < 0.05, **p < 0.01, ***p < 0.001, n. s, not significant

Next, we investigated the effects of HDAC7 in astrocyte-mediated neurotoxicity under Aβ stimulation. WT and HDAC7 knockout astrocytes were exposed to Aβ oligomers for 24 h. RT-qPCR analysis revealed that Aβ exposure led to a significant upregulation of neurotoxic reactive astrocyte marker genes, including *C3, H2-D1, H2-T23, Ggta1, Gbp2,* and *Serping1* (Fig. 5A). Notably, the genetic ablation of HDAC7 markedly attenuated the expression of these neurotoxic marker genes (Fig. 5A). The reduction in the expression of neurotoxic reactive astrocyte markers C3 and Gbp2 following HDAC7 deletion was validated at the protein level (Fig. 5B). To further elucidate the influence of these astrocytes on neurons, we collected conditioned medium (CM) from both WT and HDAC7 knockout astrocytes. After Aβ treatment, the astrocytes were thoroughly washed with PBS and subsequently transitioned to a serum-free neuronal medium. The CM were collected after 24 h of culture and used to treat mouse cortical neurons (Fig. 5C). Importantly, we observed a distinct cell death in neurons when exposure to CM from Aβ-treated WT astrocytes; however, this deleterious effect was substantially attenuated when neurons were exposed to CM from HDAC7-deficient astrocytes (Fig. 5D). Collectively, these findings demonstrate that HDAC7 is a critical regulator of Aβ-mediated astrocyte neurotoxicity.

To clarify the specific role of IKK-NF-κB signaling as a downstream effector in HDAC7-mediated astrocyte neurotoxicity, we examined the effects of IKK inhibitors on astrocytes with HDAC7 overexpression. The overexpression of HDAC7 induced a pronounced upregulation of neurotoxic reactive astrocyte marker genes (Fig. 5E), which is consistent with our *in vivo* observations. Importantly, this upregulation was effectively suppressed by treatment with IKK-16, an IKK inhibitor (Fig. 5E). Western blotting analysis further confirmed that the application of IKK inhibitor reduced the protein levels of neurotoxic reactive astrocyte markers C3 and Gbp2 in astrocytes overexpressing HDAC7 (Fig. 5F). Moreover, assessments of neuronal viability using astrocyte-CM further substantiated the ameliorative effects of IKK inhibition on HDAC7-induced neurotoxicity of astrocytes (Fig. 5G and H). Notably, non-conditioned medium and conditioned medium from control astrocytes had comparable effects on neuronal viability (Supplementary Figure S3). Taken together, these data demonstrate the necessary role of IKK activation in the HDAC7-driven astrocyte neurotoxicity.

### Knockdown of HDAC7 alleviates astrocyte neurotoxicity, neuronal loss, and cognitive deficits in APP/PS1 mice

**Fig. 6:**
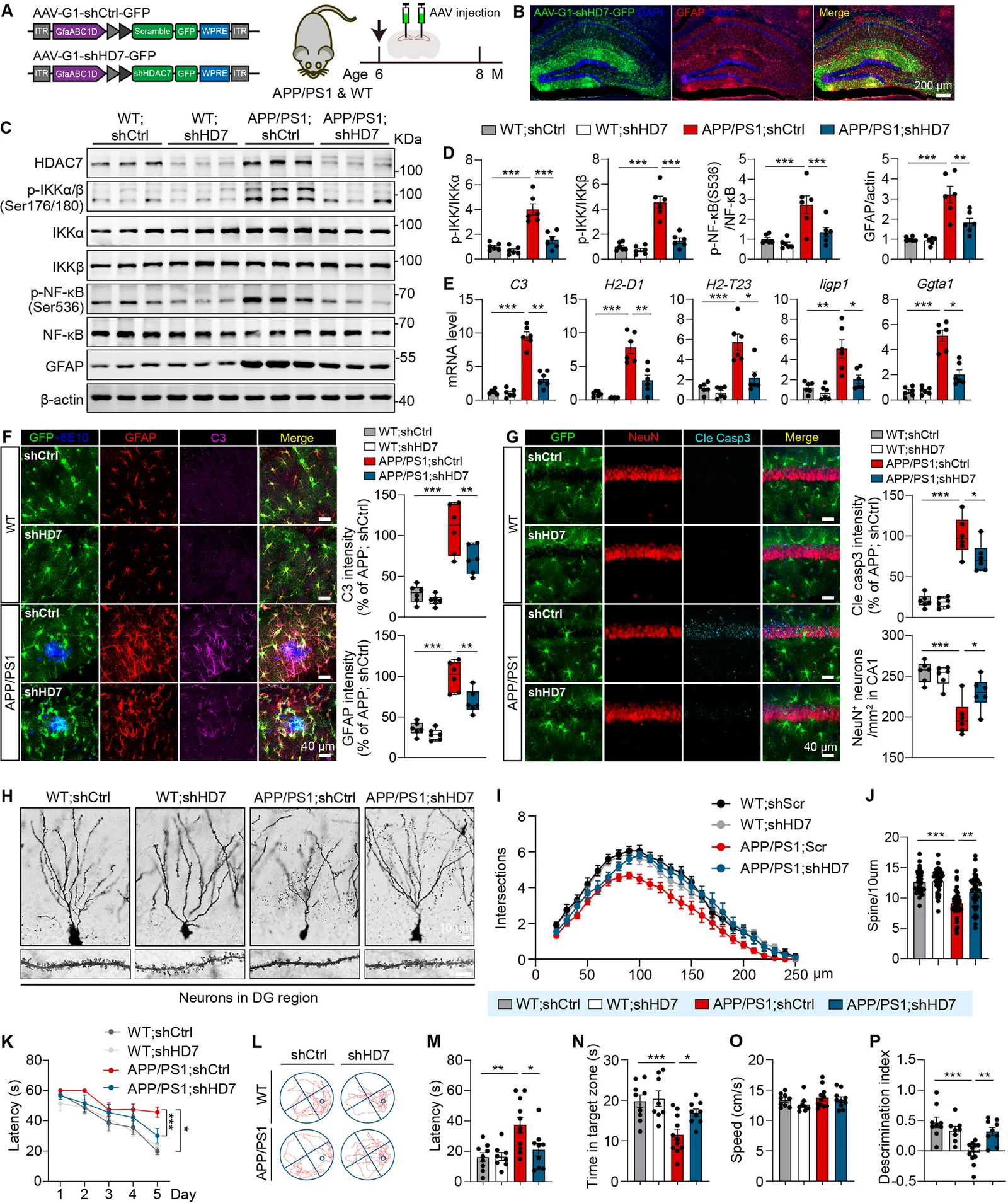
Astrocytic knockdown of HDAC7 mitigates the activation of IKK-NF-κB signaling, suppresses the formation of neurotoxic reactive astrocytes, and improves neuronal integrity and cognitive function in APP/PS1 mice. **A** Diagrammatic representation of AAVs designed with shRNA for HDAC7 knockdown or control GFP, along with the *in vivo* experimental schedule. **B** Representative immunofluorescence staining image with anti-GFAP antibody showing the targeted expression of AAV-G1-shHD7-GFP in hippocampal astrocytes of APP/PS1 mice. **C**,** D** Western blotting analysis and quantification of HDAC7, p-IKKα/β (Ser176/180), IKKα, IKKβ, p-NF-κB (Ser468), p-NF-κB (Ser536), NF-κB and GFAP in the hippocampal tissues of WT and APP/PS1 mice. n = 6 mice per group. **E** RT-qPCR analysis of neurotoxic reactive astrocyte marker genes in the hippocampal tissue samples from WT and APP/PS1 mice with astrocytic HDAC7 knockdown. n = 6 mice per group. **F** Representative co-immunostaining images of Aβ (6E10), GFAP and C3 in the hippocampal region of WT and APP/PS1 mice injected with AAVs. Quantification showing the intensity of GFAP and C3 in peri-plaque region of APP/PS1 mice or in corresponding region of WT mice. n = 6 mice per group. **G** Representative co-immunostaining images of NeuN and cleaved Caspase3 in the hippocampal CA1 region of WT and APP/PS1 mice. Quantification showing the intensity of cleaved Caspase3 and the number of NeuN^+^ neurons in hippocampal CA1 region. n = 6 mice per group. **H** Representative images of dendritic morphology and dendritic spines of neurons by Golgi staining in the hippocampus of WT and APP/PS1 mice following AAV injection. Scale bar: 10 μm. **I** Sholl analysis of dendritic intersections. n = 20–25 cells from 6 slices of 3 mice per group. **J** Quantification of spine numbers, n = 32–39 from 6 slices of 3 mice per group. **K–O** The MWM behavioral analysis. **K** Escape latency during the training phase, **L** Representative traveling traces, **M** first latency to reach the platform, **N** exploring time in target zone, and **O** swimming speed during the probe trial. **P** Discrimination index for the novel object of mice in the NOR behavioral test. For behavioral tests, n = 8–11 mice per group. Statistical significance was determined by one-way ANOVA or two-way ANOVA (**K**) with Tukey’s post hoc analysis. Data are shown as mean ± SEM, *p < 0.05, **p < 0.01, ***p < 0.001, n. s, not significant

Given the neuroprotective effects of astrocytic HDAC7 deficiency *in vitro*, we aimed to investigate the therapeutic potential of astrocytic HDAC7 deletion *in vivo*. To achieve this, we constructed AAV vectors to express a small hairpin RNA (shRNA) targeting HDAC7 (shHD7) or a scramble control shRNA (shCtrl), both driven by the GfaABC1D promoter (Fig. 6A). These AAV vectors were stereotaxically delivered into the hippocampus of 6-month-old APP/PS1 mice and age-matched WT mice (Fig. 6A). This time point is marked by a notable increase in HDAC7 levels and the formation of neurotoxic reactive astrocytes surrounding Aβ-plaque in APP/PS1 mice (Fig. 1). Two months post-injection, the targeted expression of AAV in hippocampal astrocytes was confirmed via immunostaining using an anti-GFAP antibody (Fig. 6B). The efficacy of HDAC7 knockdown in astrocytes was validated through co-immunostaining for HDAC7 and GFAP (Supplementary Figure S4A) as well as by Western blotting analysis (Fig. 6C). Western blotting analysis in hippocampal tissues demonstrated that the knockdown of HDAC7 led to reduced activation of the IKK-NF-κB signaling pathway and a decrease in GFAP levels in APP/PS1 mice (Fig. 6C and D). Subsequently, we assessed the expression levels of genes encoding neurotoxic reactive astrocytes (*C3, H2-D1, H2-T23, Iigp1* and *Ggta1*) in the brain of WT and APP/PS1 mice subjected to AAV injection. RT-qPCR analysis revealed that the induction of these marker genes in the hippocampus of APP/PS1 mice was significantly mitigated by HDAC7 knockdown in astrocytes (Fig. 6E). To further elucidate the characteristics of neurotoxic reactive astrocytes, we conducted immunofluorescence co-staining for Aβ (6E10), pan-reactive astrocyte marker (GFAP) and neurotoxic marker (C3). Our findings demonstrated that the expression levels of GFAP and C3 in the 6E10^+^ plaque-adjacent regions of APP/PS1 mice was dramatically reduced by HDAC7 knockdown (Fig. 6F), indicating a decrease in neurotoxic reactive astrocytes. Importantly, upon astrocytic HDAC7 knockdown, while the expression level of C1q was reduced, the expression levels of Il-1α and Tnfα remained unaffected (Supplementary Figure S4B). This suggests that HDAC7 knockdown modulates the functions of neurotoxic reactive astrocytes more directly within astrocytes rather than through microglial interactions.

We then assessed the effects of astrocytic HDAC7 downregulation on neurons. Immunofluorescence co-staining revealed that the knockdown of HDAC7 in astrocytes resulted in a decrease in cleaved Caspase3 activation and mitigated the loss of NeuN^+^ neurons within the hippocampus of APP/PS1 mice, indicating improved neuronal survival associated with astrocytic HDAC7 deficiency (Fig. 6G). Further Golgi staining analysis indicated that astrocytic HDAC7 knockdown reinstated dendritic complexity and increased the number of spines in hippocampal neurons of APP/PS1 mice (Fig. 6H-J). Collectively, these findings imply that the absence of astrocytic HDAC7 can reduce the formation of neurotoxic reactive astrocytes, thereby promoting neuronal survival and synaptic plasticity in the APP/PS1 mouse model.

The MWM test was employed to evaluate hippocampus-dependent spatial learning and memory. In comparison to control WT mice, APP/PS1 mice injected with shCtrl exhibited significant impairments in spatial learning and memory, as evidenced by increased escape latency and reduced exploring time in the target zone (Fig. 6K-N). Notably, these behavioral deficits were l substantially ameliorated in APP/PS1 mice that were injected with shHD7 (Fig. 6K-N). Swimming speed was consistent across all experimental groups (Fig. 6O). Furthermore, APP/PS1 mice injected with shCtrl demonstrated a decreased preference for the novel object in the NOR test, which was not observed in APP/PS1 mice injected with shHD7 (Fig. 6P), indicating improved memory function.

The impact of HDAC7 downregulation in astrocytes on Aβ pathology was also assessed. Immunostaining with the anti-6E10 antibody revealed a significant reduction in Aβ burden within the hippocampus of APP/PS1 mice injected with shHD7, compared to those treated with shCtrl (Supplementary Figure S4C). Further ELISA analysis demonstrated a substantial decrease in both soluble and insoluble Aβ_40_ and Aβ_42_ levels in the hippocampus of shHD7-injected APP/PS1 mice (Supplementary Figure S4D).

Collectively, the restoration of hippocampus-dependent cognitive function, coupled with our histopathological findings of enhanced neuronal survival and reduced Aβ pathology, supports the notion that targeting HDAC7 in astrocytes leads to an amelioration of neuropathology and cognitive enhancement in a mouse model of AD.

### HDAC7 inhibition with TMP195 suppresses NF-κB signaling activation and reduces peri-plaque neurotoxic reactive astrocytes in APP/PS1 mice

**Fig. 7:**
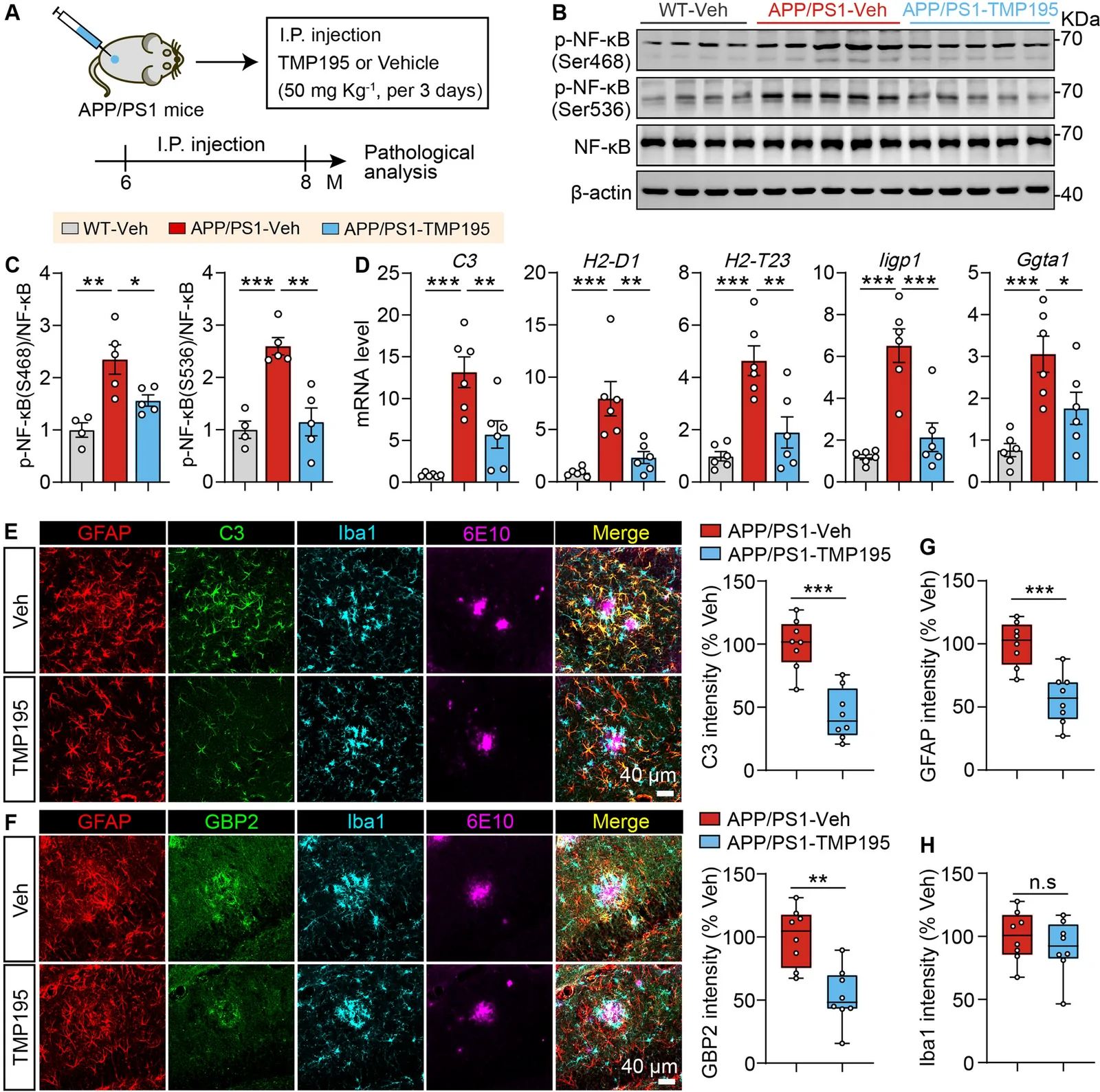
HDAC7 inhibition with TMP195 suppresses NF-κB signaling activation and plaque-associated neurotoxic astrocytes in APP/PS1 mice. **A** A schematic diagram shows the *in vivo* administration of TMP195 in mice. **B**,** C** Western blotting analysis and quantification of p-NF-κB (Ser468), p-NF-κB (Ser536), and NF-κB in the hippocampal tissues of WT and APP/PS1 mice administrated with TMP195 or vehicle. n = 4–5 mice per group. **D** RT-qPCR analysis of neurotoxic reactive astrocyte marker genes in the hippocampal tissue samples from mice administrated with TMP195 or vehicle. n = 6 mice per group. **E, F** Representative co-immunostaining images of Aβ (6E10), Iba1, GFAP and **E** C3 or **F** GBP2 in the hippocampus of APP/PS1 mice treated with TMP195 or vehicle. Quantification showing the intensity of C3 and GBP2 in peri-plaque region in APP/PS1 mice. n = 8 mice per group. **G, H** Quantification of the intensity of GFAP and Iba1 in peri-plaque region in APP/PS1 mice. n = 8 mice per group. Statistical significance was determined by one-way ANOVA with Tukey’s post hoc analysis (**C**, **D**) or unpaired Student’s t test (**E**–**H**). Data are shown as mean ± SEM, *p < 0.05, **p < 0.01, ***p < 0.001, n. s, not significant

In light of the observed beneficial effects of astrocytic HDAC7 deficiency in APP/PS1 mice, we proceeded to evaluate the therapeutic potential of HDAC7 pharmacological inhibition. The selective class IIa HDAC inhibitor, TMP195, has been extensively characterized for its ability to inhibit HDAC7 activity within the brain, while exhibiting minimal toxicity to peripheral organs when administered via intraperitoneal injection [29]. Accordingly, TMP195 was administered intraperitoneally to 6-month-old APP/PS1 mice at a dosage of 50 mg/kg every three days over a two-month period, with age-matched WT mice receiving vehicle injections as controls (Fig. 7A). Consistent with the findings, western blotting analysis demonstrated that TMP195 treatment resulted in decreased phosphorylation levels of NF-κB at Ser468 and Ser536 in the hippocampus of APP/PS1 mice (Fig. 7B and C). RT-qPCR analysis of *C3, H2-D1, H2-T23, Iigp1* and *Ggta1* revealed a broadly diminished neurotoxic gene signature of astrocytes in the hippocampus of APP/PS1 mouse administered with TMP195 (Fig. 7D). To further explore the development of neurotoxic reactive astrocytes in the peri-plaque region, we performed immunofluorescence co-immunostaining for Aβ (6E10), microglia (Iba1), pan-reactive astrocyte maker (GFAP), and neurotoxic astrocyte markers (C3 or GBP2) in brain sections from APP/PS1 mice. The findings indicated that TMP195 treatment significantly reduced the expression of neurotoxic markers C3 and GBP2, as well as the pan-reactive marker GFAP, in the 6E10^+^-plaque-adjacent area (Fig. 7E-G), indicating a reduction in neurotoxic reactive astrocytes. Notably, the expression levels of Il-1α, TNFα, and C1q (Supplementary Figure S5), along with the intensity of Iba1 (Fig. 7H), were unaffected by TMP195 treatment. These results imply that the beneficial effects of TMP195 are predominantly mediated through its direct action on astrocytes rather than through microglial pathways.

### HDAC7 inhibition alleviates neuronal loss, cognitive deficits and Aβ burden in APP/PS1 mice

**Fig. 8:**
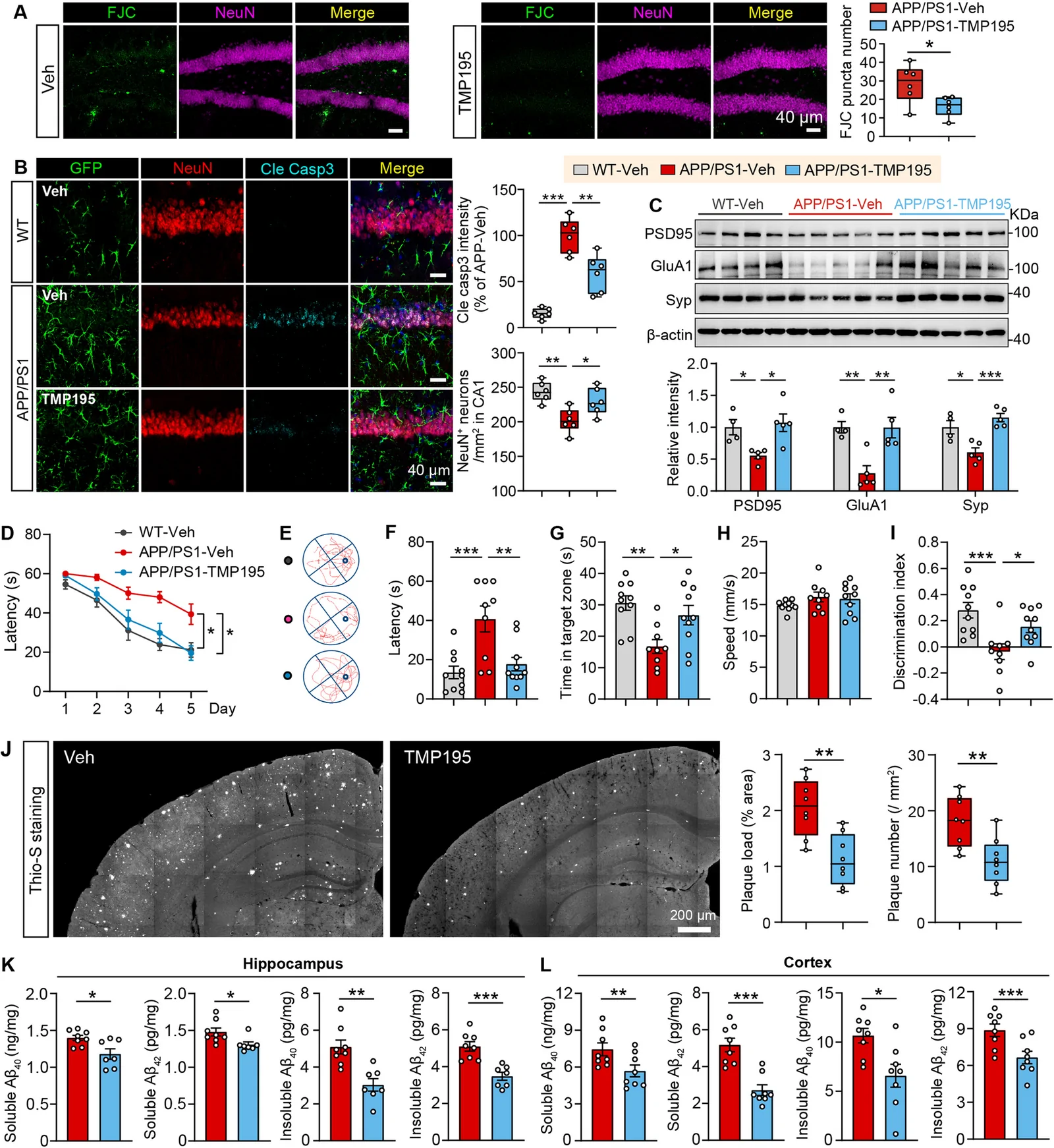
HDAC7 inhibition with TMP195 ameliorates neurodegeneration, improves cognitive function, and reduces Aβ burden in APP/PS1 mice. **A** Representative images of Fluoro Jade C (FJC) staining combined with anti-NeuN immunostaining in the hippocampal DG from APP/PS1 mice treated with TMP195 or vehicle. The number of FJC puncta in the DG area was quantified, n = 6 mice per group. **B** Representative co-immunostaining images of cleaved Caspase3, NeuN, and GFAP in the hippocampal CA1 region of WT and APP/PS1 mice treated with TMP195 or vehicle. Quantification showing the intensity of cleaved Caspase3 and the number of NeuN^+^ neurons in hippocampal CA1 region. n = 6 mice per group. **C** Western blotting analysis and quantification of synaptic proteins PSD95, GluA1, and Synaptophysin (Syp) in the hippocampal samples of mice treated with TMP195 or vehicle. n = 4–5 mice per group. **D-H** MWM behavioral test analysis of mice treated with TMP195 or vehicle. **D** Escape latency during the training phase, **E** Representative traveling traces, **F** first latency to reach the platform region, **G** exploring time in target zone, and **H** swimming speed during the probe trial. **I** Discrimination index of mice treated with TMP195 or vehicle in the NOR behavioral test. For behavioral tests of TMP195 administration, n = 9–10 mice per group. **J** Representative images of Thioflavin-S staining in the brain sections from APP/PS1 mice treated with TMP195 or vehicle. The load area and number of Thio-S^+^ plaques was quantified. **K**,** L** ELISA analysis of soluble and insoluble Aβ_40_ and Aβ_42_ in the hippocampal and cortical tissues from APP/PS1 mice treated with TMP195 or vehicle. Statistical significance was determined by unpaired Student’s t test (**A**, **J**, **K** and **L**) or one-way ANOVA (**B**, **C**, **F**-**I**) or two-way ANOVA (**D**) with Tukey’s post hoc analysis. Data are shown as mean ± SEM, *p < 0.05, **p < 0.01, ***p < 0.001, n. s, not significant

Next, we explored the neuronal and cognitive effects of HDAC7 inhibition in APP/PS1 mice. Neuronal degeneration in the hippocampus was evaluated using Fluoro-Jade C (FJC) staining, a method that specifically binds to and visualizes damaged neurons. The results from FJC staining demonstrated a significant reduction in the number of FJC-positive puncta was substantially reduced in the hippocampus of APP/PS1 mice treated with TMP195 (Fig. 8A). Further immunofluorescence co-staining analysis demonstrated that TMP195 treatment decreased the levels of cleaved Caspase3 and mitigated the loss of NeuN^+^ neurons within the hippocampus of APP/PS1 mice (Fig. 8B), suggesting improved neuronal survival following TMP195 treatment. These findings were further corroborated by Western blotting analysis, which revealed that TMP195 treatment restored the levels of synaptic-associated proteins, including PSD95, GluA1 and synaptophysin (Fig. 8C). Collectively, these data suggest that HDAC7 inhibition via TMP195 attenuates neuronal damage *in vivo*. We further evaluated the therapeutic effects of TMP195 on the cognitive performance of APP/PS1 mice. In the MWM behavioral test, APP/PS1 mice treated with TMP195 exhibited an accelerated learning trajectory during the training phase, comparable to that of WT mice (Fig. 8D). During the probe phase, TMP195-treated mice showed improved memory performance, as evidenced by reduced latency to reach the platform, increased exploring time in target zone, and more frequent crossings over the platform region (Fig. 8E-G), while maintaining comparable swimming speeds across all groups (Fig. 8H). The improvement in cognitive function was further corroborated by the NOR test, wherein the TMP195-treated animals displayed a pronounced preference for the new object introduced during the probe session (Fig. 8I).

The impact of TMP195 on Aβ burden in APP/PS1 mice was also assessed. Thioflavin-S staining analysis of brain sections revealed a marked reduction in both the number and load area of Thio-S positive plaques in APP/PS1 mice treated with TMP195 (Fig. 8J). Furthermore, we quantified the levels of soluble and insoluble Aβ peptides in the hippocampal and cortical tissue samples of APP/PS1 mice. ELISA analysis indicated a significant decrease in the levels of both Aβ_40_ and Aβ_42_ species in the hippocampus and cortex following TMP195 administration (Fig. 8K and L).

Collectively, our findings indicate that HDAC7 inhibition can reduce the gene expression and functional activities of neurotoxic reactive astrocytes, thereby attenuating neuronal loss, reducing Aβ burden and improving cognitive deficits in APP/PS1 mice.

## Discussion

The conversion of astrocytes into a reactive state is a prominent characteristic of neuropathology [2]. Nevertheless, the molecular mechanisms that dictate whether astrocytes assume neurotoxic or neuroprotective phenotypes remain inadequately elucidated, particularly in the context of AD [8, 14, 42]. In this study, we identify astrocytic HDAC7 as an effector of Aβ pathology, which promotes the development of neurotoxic reactive astrocytes via direct deacetylation and activation of IKKα/β, consequently leading to NF-κB-dependent transcription of neurotoxic genes. Through astrocyte-specific gain- and loss-of-function approaches in APP/PS1 mice, we demonstrate that HDAC7 is sufficient for the induction of astrocyte-mediated neurotoxicity, neuronal loss, and cognitive decline. Furthermore, pharmacological inhibition of HDAC7 using TMP195 replicates the protective effects observed with HDAC7 knockdown, mitigating peri-plaque neurotoxic astrocytes and ameliorating neuropathology. These findings establish HDAC7 as a mediator linking Aβ pathology to astrocyte-driven neurodegeneration.

A dominant model in the field posits that neurotoxic reactive astrocytes are primarily induced by microglia-derived cytokines IL-1α, TNF-α, and C1q [6, 10]. Our study challenges the exclusivity of this model by demonstrating that astrocytic HDAC7 upregulation is sufficient to drive a neurotoxic transcriptional program and neuronal death without inducing significant changes in the expression of these three microglia-derived cytokines. Conversely, HDAC7 inhibition reduces the presence of peri-plaque neurotoxic reactive astrocytes without affecting microglial activation or the expression of these three cytokines. These findings support an emerging perspective that astrocytes can adopt neurotoxic phenotypes through cell-autonomous mechanisms, independent of microglial influence. This aligns with recent studies indicating that astrocyte-specific overexpression of growth arrest and DNA damage inducible gamma or direct exposure to tau fibrils can similarly induce neurotoxic states [19, 43]. The progressive upregulation of HDAC7 in plaque-adjacent astrocytes with advancing disease stage, along with our observation that Aβ oligomers directly increase HDAC7 expression in cultured astrocytes, suggests that Aβ itself may serve as an upstream trigger for this intrinsic pathway. The exact mechanism by which extracellular Aβ induces HDAC7 upregulation in astrocytes has yet to be fully elucidated. Previous studies have demonstrated that the *HDAC7* promoter contains a functional specificity protein 1 (Sp1) binding site, which is crucial for its transcriptional activation in response to extracellular stimuli [44]. Sp1 is known to be activated in the brain of AD patients, and Aβ has been reported to activate Sp1 in cultured cells [45, 46]. Therefore, it is plausible that Aβ may facilitate HDAC7 transcription via an Sp1-dependent pathway, warranting further investigation. Importantly, elevated HDAC7 levels in astrocytes has also been observed in tauopathy models [29], indicating the potential role of HDAC7 as a shared effector mechanism across distinct proteinopathies.

The IKK-NF-κB signaling axis is a well-established mediator of inflammatory responses in astrocytes [15]. Our mechanistic studies have elucidated that HDAC7 activates this pathway via a previously unrecognized post-translational mechanism: the direct deacetylation of IKKα and IKKβ. Co-immunoprecipitation experiments in primary astrocytes demonstrate that HDAC7 interacts with IKKα/β following Aβ stimulation, resulting in reduced acetylation of these kinases and enhanced IKK activity, as evidenced by increased phosphorylation of NF-κB p65 at Ser468 and Ser536. This mechanism is consistent with previous findings in other cell systems, where class IIa HDACs are known to regulate IKK activity through deacetylation [47, 48]. Importantly, HDAC7 deletion or pharmacological inhibition restores the acetylation of IKKα/β and attenuates downstream NF-κB activation and C3 expression. While our findings definitively position HDAC7 as an upstream regulator of IKK, the specific acetylation sites on IKKα/β that facilitate this regulation remain to be identified. Furthermore, whether HDAC7 differentially modulates IKKα versus IKKβ, and whether additional downstream pathways contribute to the neurotoxic phenotype, are critical questions that warrant further investigation.

One unexpected finding in this study is that the knockdown or pharmacological inhibition of HDAC7 leads to a reduction of amyloid plaque burden in APP/PS1 mice. This observation raises mechanistic inquiries regarding the interplay between astrocyte reactivity and Aβ pathology. The dual role of astrocytes in AD is well recognized: reactive astrocytes can promote Aβ clearance, but prolonged inflammatory activation may exacerbate plaque deposition and neuronal injury [19, 42, 49, 50]. Our data suggest two non-mutually exclusive possibilities. Firstly, by preserving neuronal health, HDAC7 inhibition may indirectly diminish the primary source of Aβ production. Secondly, considering our previous finding that HDAC7 deficiency enhances autophagy-lysosomal function in tauopathy models [29], HDAC7 inhibition may directly enhance astrocytic Aβ clearance. These possibilities are supported by studies indicating that attenuation of astrocyte activation through *Stat3* or *Chi3l1* knockout reduces plaque burden in APP/PS1 mice [51, 52]. Further investigation employing cell-type-specific manipulation of HDAC7, combined with direct monitoring of Aβ production and degradation, may elucidate the distinct mechanisms involved.

Sustained astrocytic IKK activation has been reported to reduce Aβ deposition in certain studies [53]. IKK phosphorylates multiple NF-κB-independent substrates that regulate autophagy and phagocytic clearance, pathways critically involved in Aβ degradation. For example, IKKα phosphorylates p53 to transactivate the autophagic mediator DRAM1 [54]; IKKα also regulates AMBRA1 phosphorylation to promote mitophagy [55]. Additionally, IKKβ phosphorylates SNAP23 to facilitate autophagosome-lysosome fusion [56]. Notably, post-translational modifications of IKK, such as ubiquitination, have been shown to affect its substrate recognition [57]. Consequently, distinct modification states, such as acetylation in our study versus other modifications in different contexts, may determine its substrate selectivity and direct the kinase toward either neuroprotective (autophagy-promoting) or neurotoxic (NF-κB-driven) outcomes. This context-dependent perspective is further supported by recent findings, which demonstrate that HDAC inhibitors exert varying effects on glial cells depending on dosage and experimental paradigm [58]. Additionally, the HDAC6 inhibitor LASSBio-1911 has been shown to reduce astrocyte reactivity and cognitive deficits in an Aβ oligomer model [59], supporting the therapeutic potential of class II HDAC inhibition in AD.

### Limitations

Several limitations of this study warrant acknowledgment, alongside potential future research directions aimed at addressing these gaps. Firstly, while we have identified HDAC7-mediated deacetylation of IKKα/β as a key mechanism, the specific acetylation sites that regulates IKK activity remain unidentified. Our mechanistic investigations were primarily conducted in primary astrocytes, which may not fully recapitulate the complexity of glial interactions *in vivo*. Additionally, our bulk RNA-seq data derived from hippocampal tissues may be impacted by variations in cell type proportions, including reactive gliosis or neuronal loss. While our data support astrocyte-specific effects, future studies utilizing acetylation site mutagenesis, conditional knockout mice, and single-cell transcriptomics are necessary to confirm the cell-autonomous role of HDAC7 and to elucidate cell-type-specific transcriptional responses. Secondly, all *in vivo* experiments were performed using the APP/PS1 transgenic mouse model, which overexpresses mutant human amyloid precursor protein and presenilin-1. While this model replicates certain aspects of familial AD, it does not encapsulate the full complexity and heterogeneity of sporadic AD, particularly with respect to tau pathology. Consequently, this limitation affects the translational relevance of our findings. Thirdly, we initiated HDAC7 inhibition at 6 months of age in APP/PS1 mice, a stage characterized by moderate cognitive deficits [60, 61]. However, we did not evaluate the treatment efficacy at more advanced stages of the disease (e.g., 8–10 months). It remains uncertain whether HDAC7 inhibition is effective when initiated at later stages of Aβ pathology. Future studies employing staggered initiation treatment paradigms would enhances the translational potential of targeting HDAC7 inhibition. Fourthly, TMP195 is a selective class IIa HDAC inhibitor but does not distinguish among HDAC4, 5, 7, and 9. Thus, we cannot fully exclude contributions from other class IIa family members to the observed neuroprotective effects. This limitation is commonly encountered in studies employing pan-class IIa HDAC inhibitors. Nevertheless, the concordance between knockdown and pharmacological inhibition, alongside direct biochemical evidence of HDAC7-IKK interaction, robustly supports a pivotal role for HDAC7. The future development of HDAC7-selective small-molecule inhibitors would enhance therapeutic precision. Looking ahead, beyond these mechanistic and experimental limitations, the therapeutic potential of targeting HDAC7 could be advanced through the development of astrocyte-targeted drug delivery systems or HDAC7-selective small molecules. Considering that HDAC7 upregulation has also been documented in tauopathy models [29], such strategies may have broader applicability across multiple neurodegenerative diseases characterized by reactive astrogliosis, including frontotemporal dementia and Parkinson’s disease.

## Conclusion

In summary, this study identifies HDAC7 as an intrinsic regulator that drives the formation of neurotoxic reactive astrocytes in response to Aβ pathology, thus contributing to neurodegeneration in AD pathogenesis. The underlying mechanism involves HDAC7-induced activation of the IKK-NF-κB pathway, through directly modulating the acetylating status of IKKα and IKKβ upon Aβ stimulation. Knockdown or pharmacological targeting of HDAC7 suppresses this pathway, reduces the formation of plaque-associated neurotoxic reactive astrocytes, and alleviates cognitive decline in APP/PS1 mice. These findings establish HDAC7 as a critical node linking Aβ accumulation to astrocyte-mediated neurodegeneration and highlight the potential of astrocyte-directed HDAC7 inhibition as a therapeutic strategy for AD and related neurodegenerative diseases.

## Supplementary Information

Supplementary Material 1.

Supplementary Material 2.

Supplementary Material 3.

## Data Availability

All data generated during this study and supporting the present results are available from the corresponding author upon reasonable request.

## Abbreviations

HDAC: Histone deacetylase
AD: Alzheimer’s disease
Aβ: Amyloid‐β
IKK: Inhibitory κB kinase
NF-κB: Nuclear factor κB
TNF: Tumor necrosis factor
NeuN: Neuronal nuclei
GFAP: Glial fibrillary acidic protein
Iba1: Ionized calcium-binding adaptor molecule 1
MMW: Morris water maze
NOR: Novel object recognition
C3: Complement C3
DEGs: Differentially expressed genes
CM: Conditioned medium
