# Supplementary material for: HDAC7 acts as an astrocytic mediator of Aβ pathology that directly engages IKK to drive astrocyte neurotoxicity and neurodegeneration in Alzheimer’s disease
Source: Alzheimers Res Ther. 2026 Jun 22;18:196. doi: 10.1186/s13195-026-02121-5 (PMC13540850; doi:10.1186/s13195-026-02121-5)

Figure 1

Figure 1B

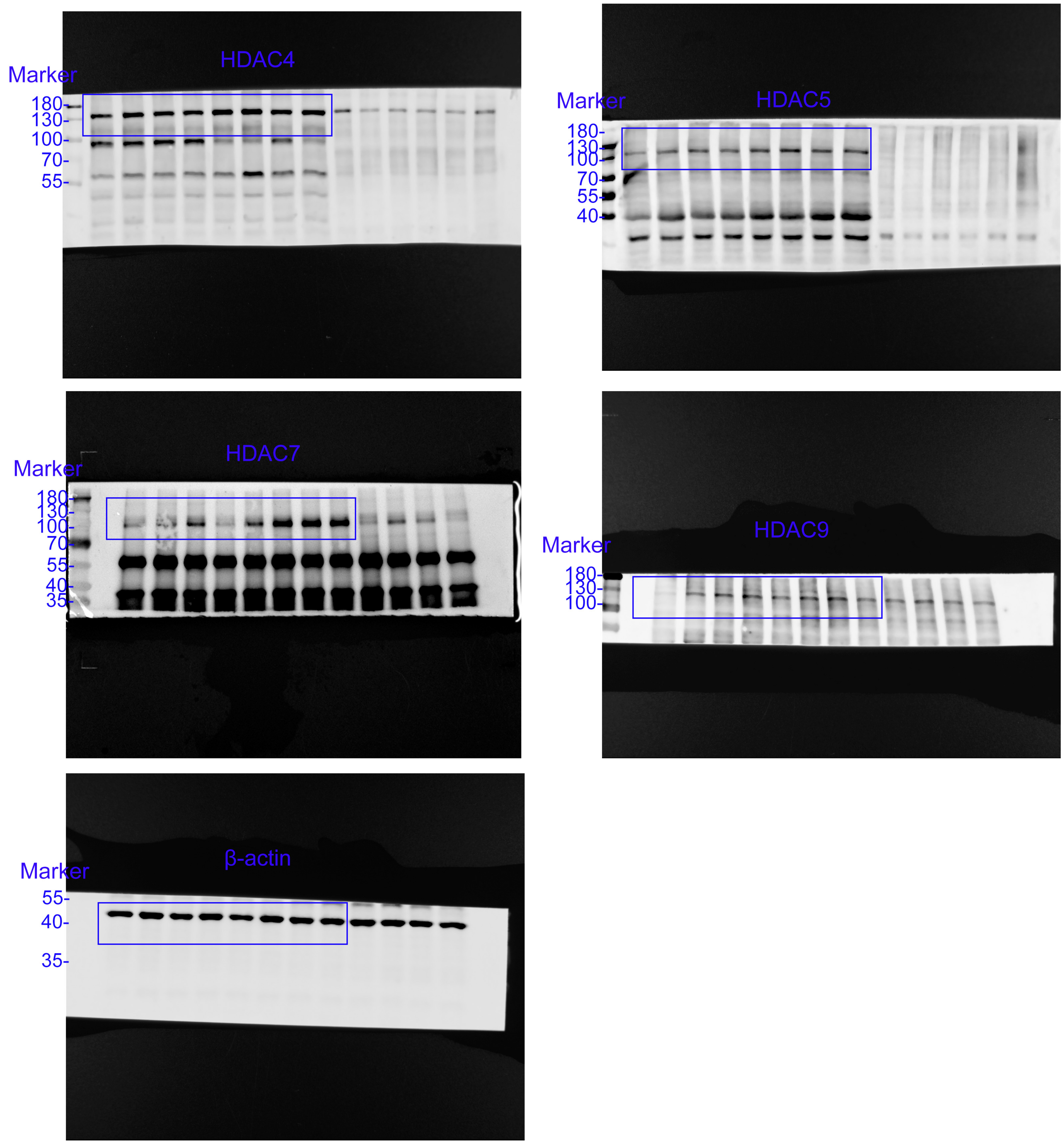

Figure 1F

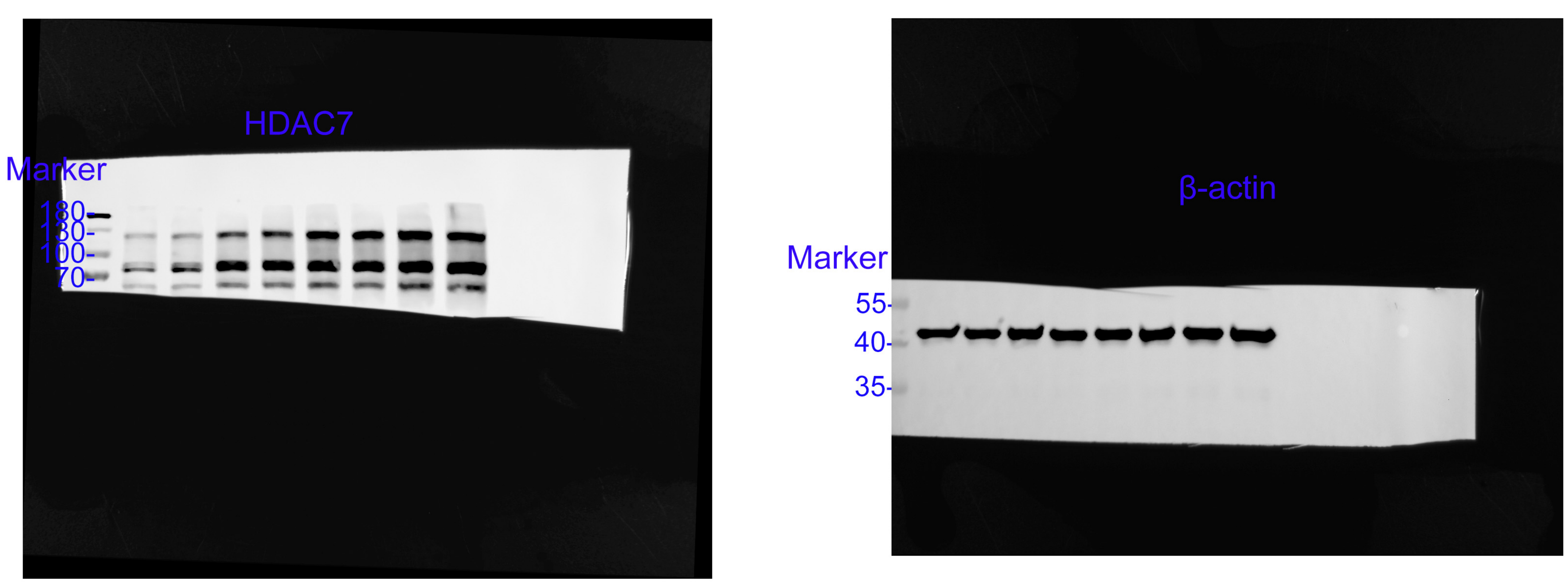

Figure 3

Figure 3G

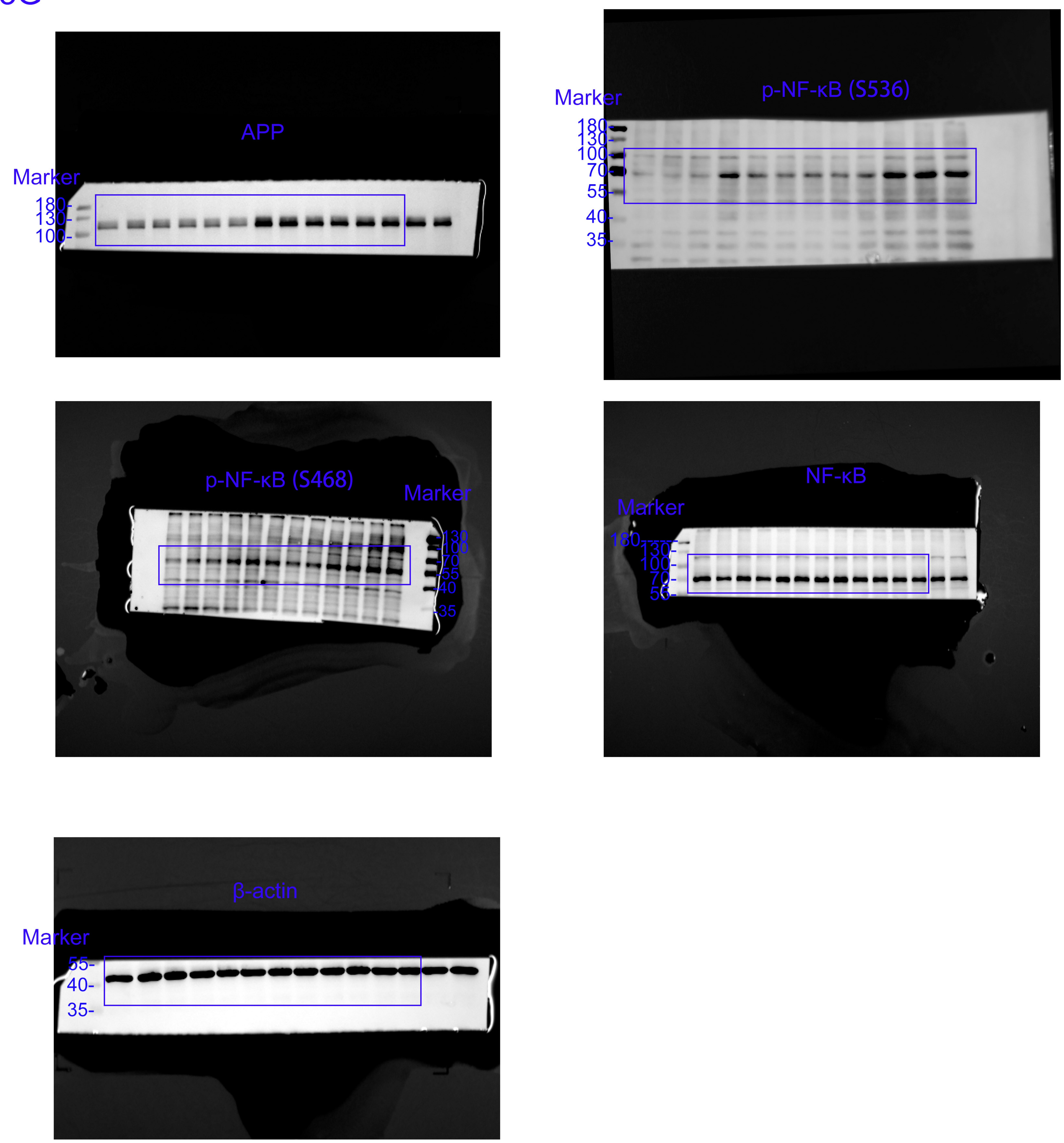

Figure 4

Figure 4A

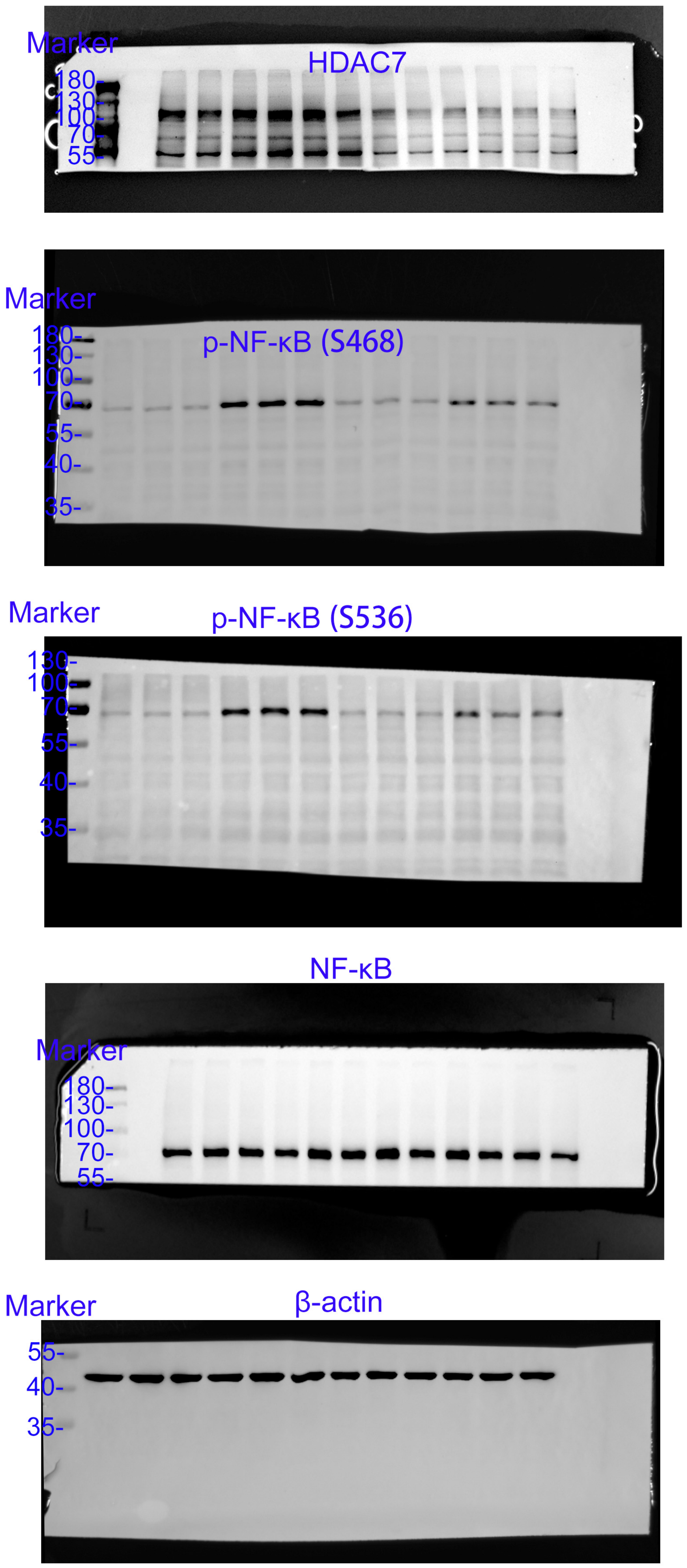

Figure 4C

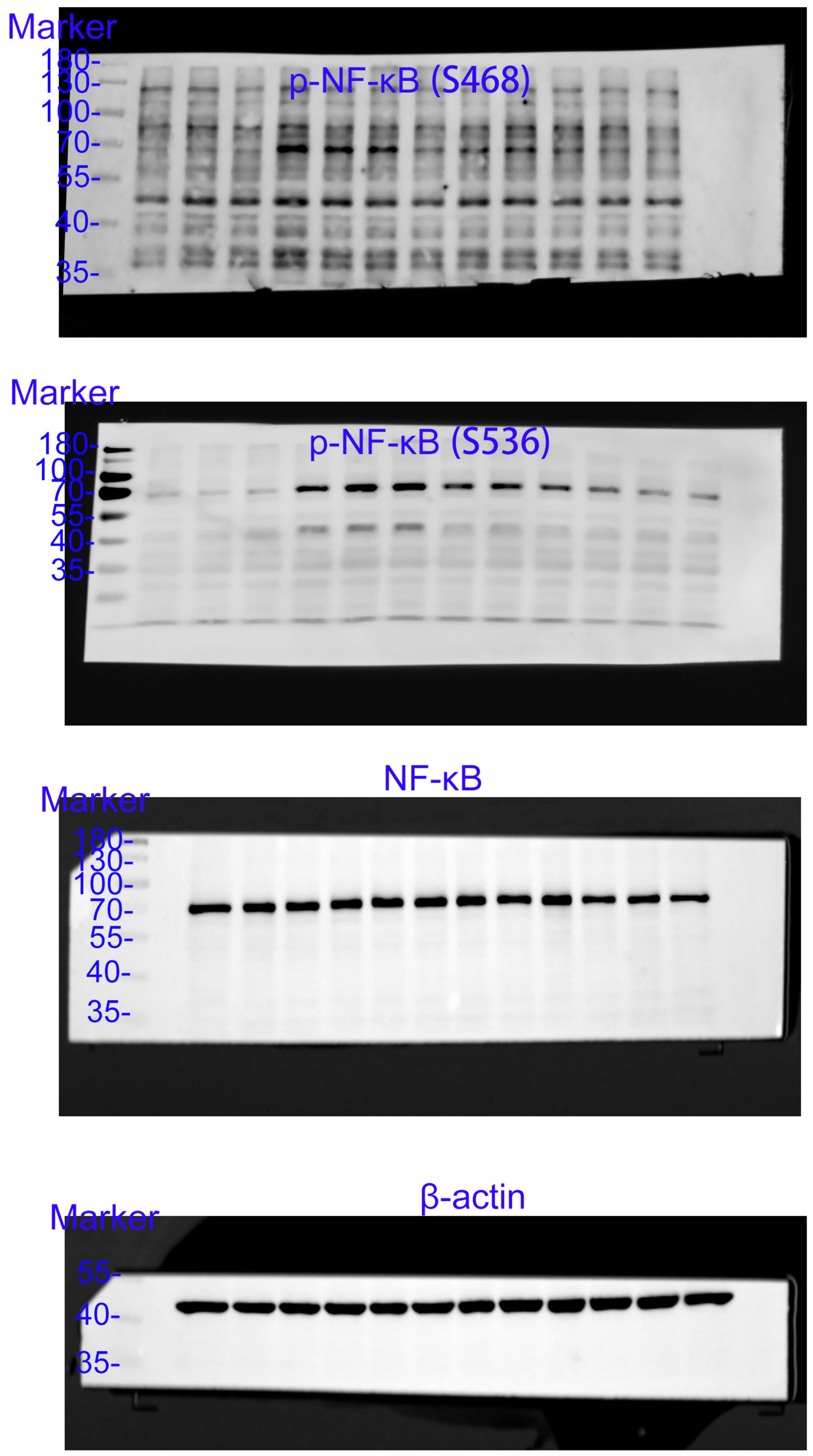

Figure 4G

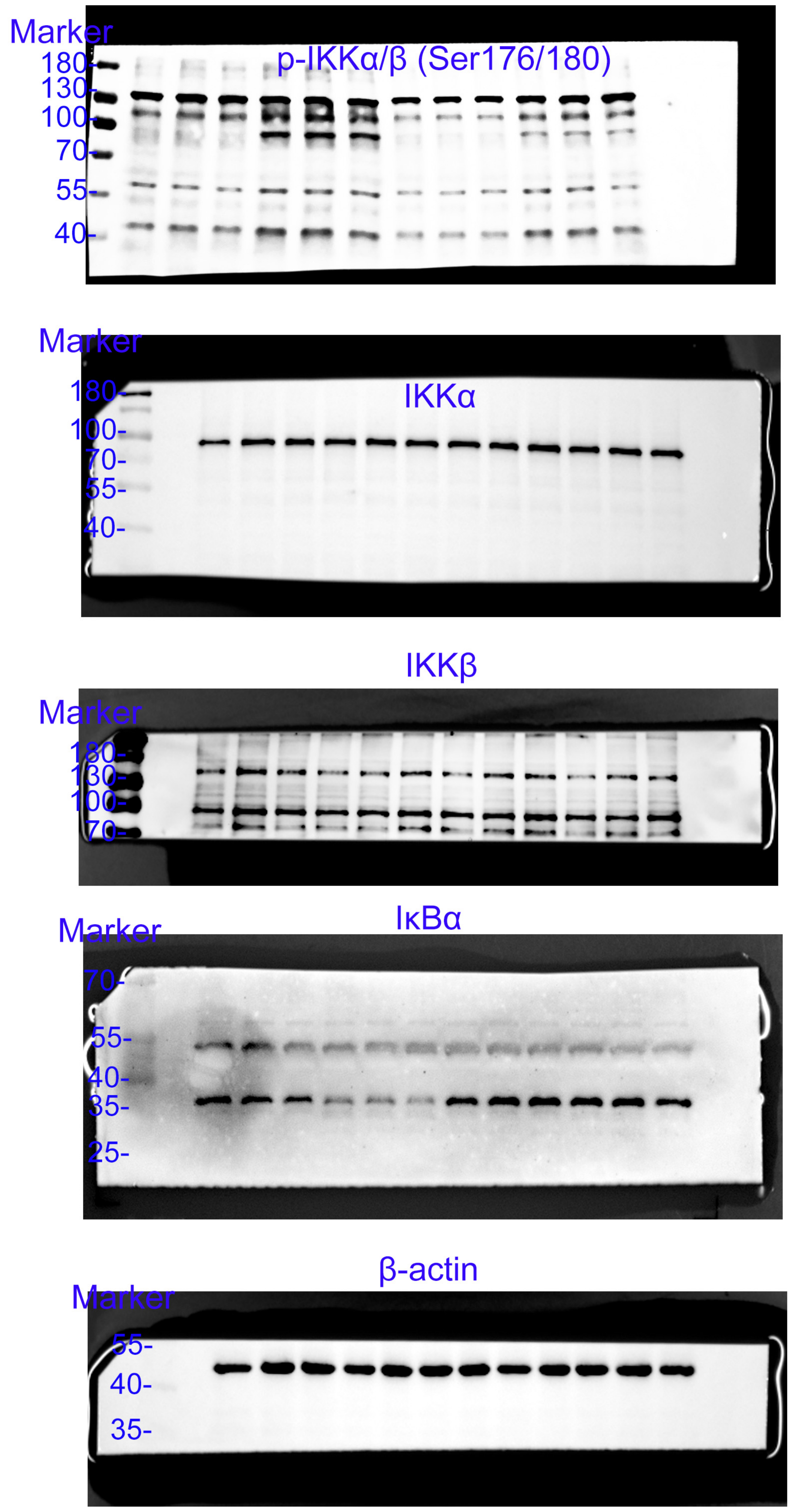

Figure 4

Figure 4I

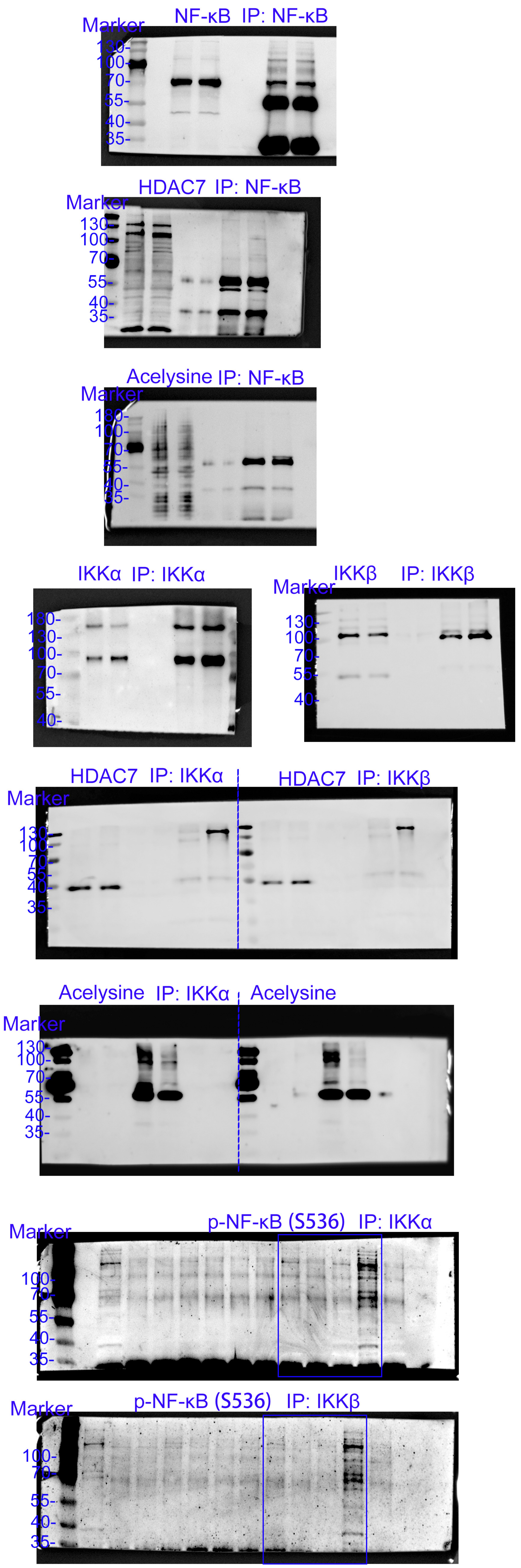

Figure 4, left panel

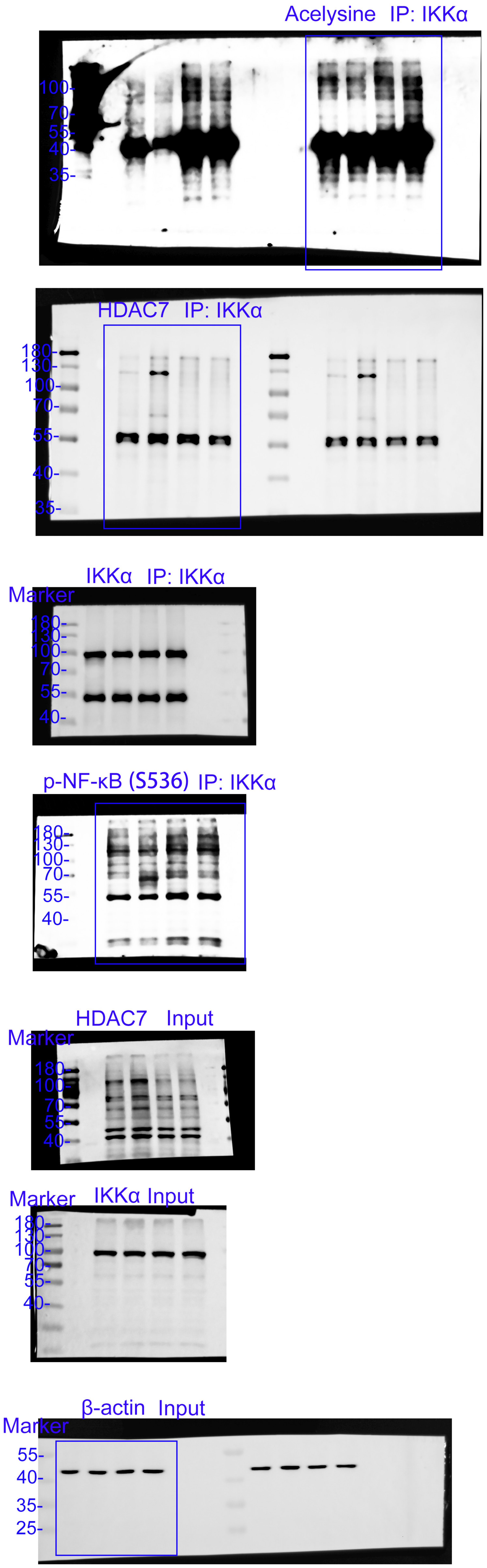

Figure 4, right panel

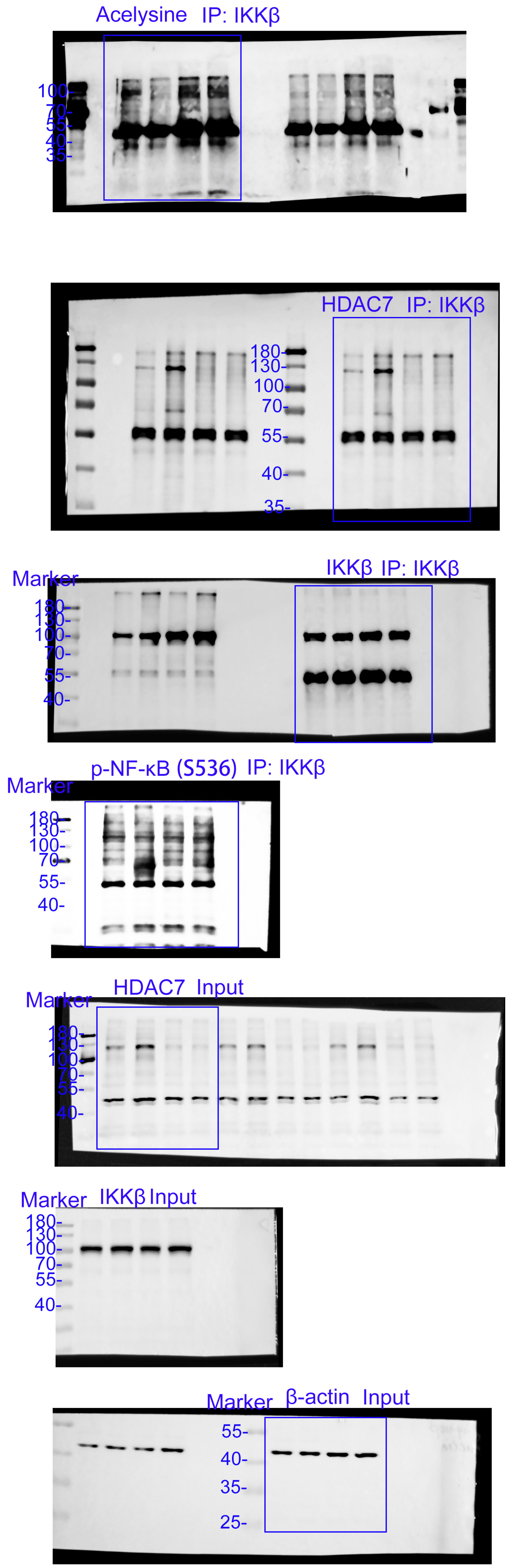

Figure 4

Figure 4K

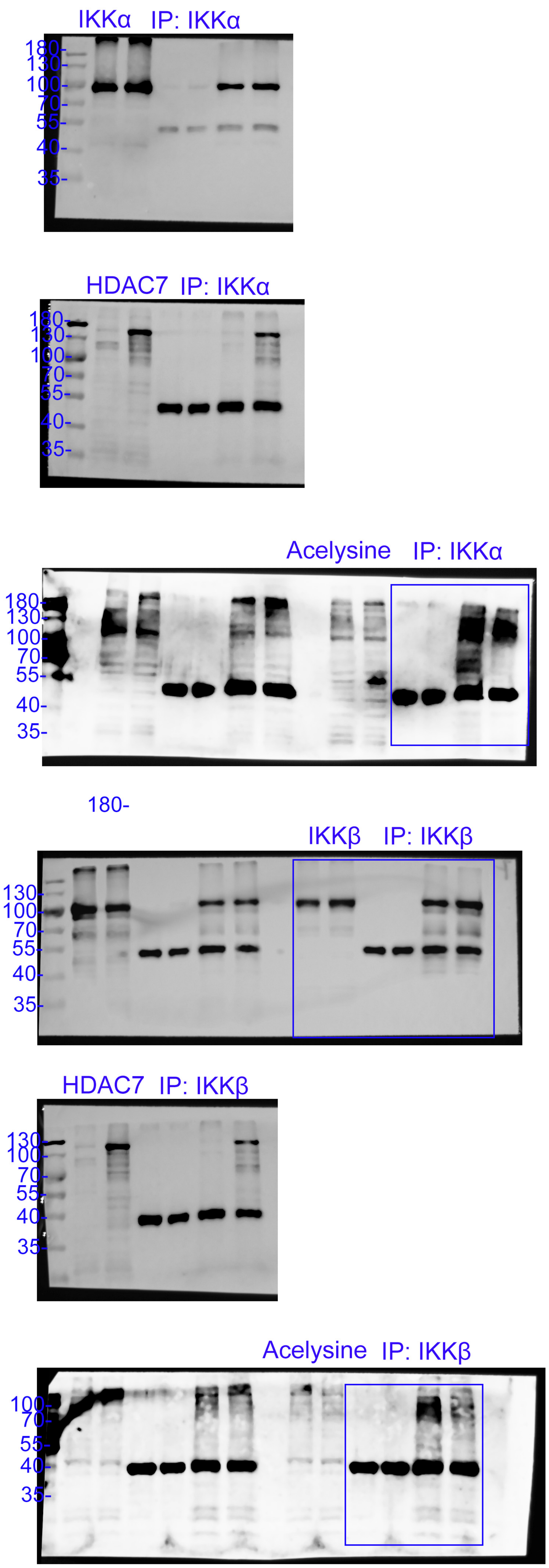

Figure 4L

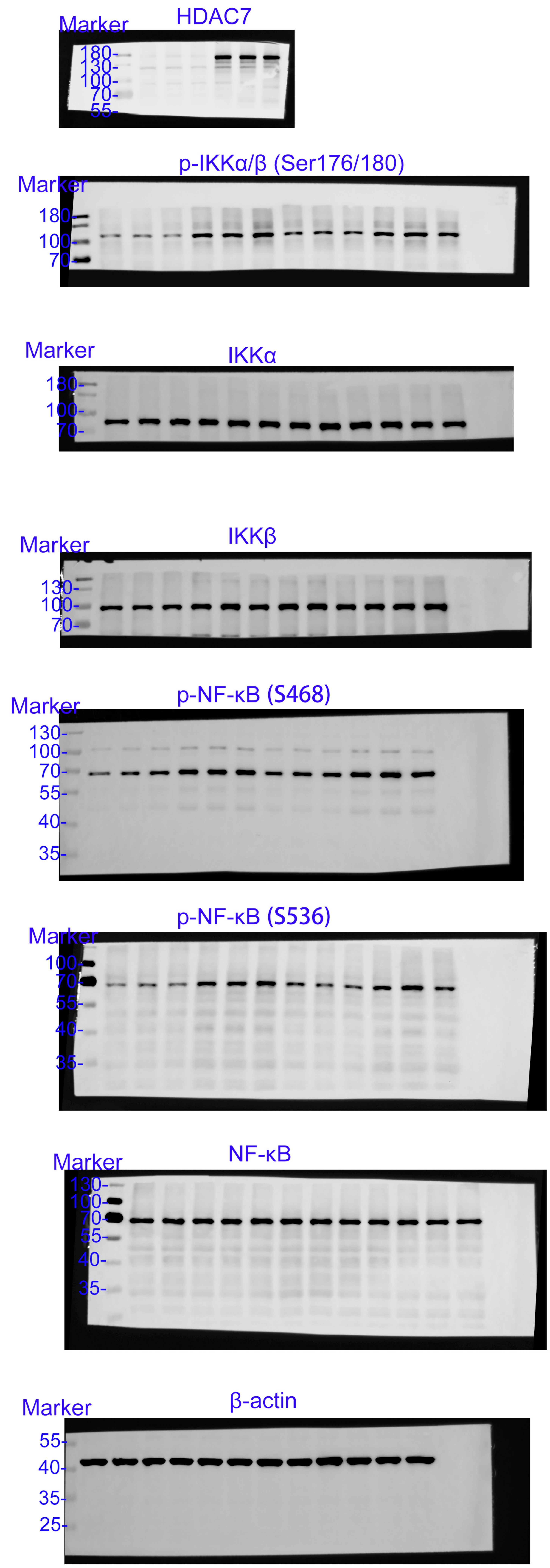

Figure 5

Figure 5B

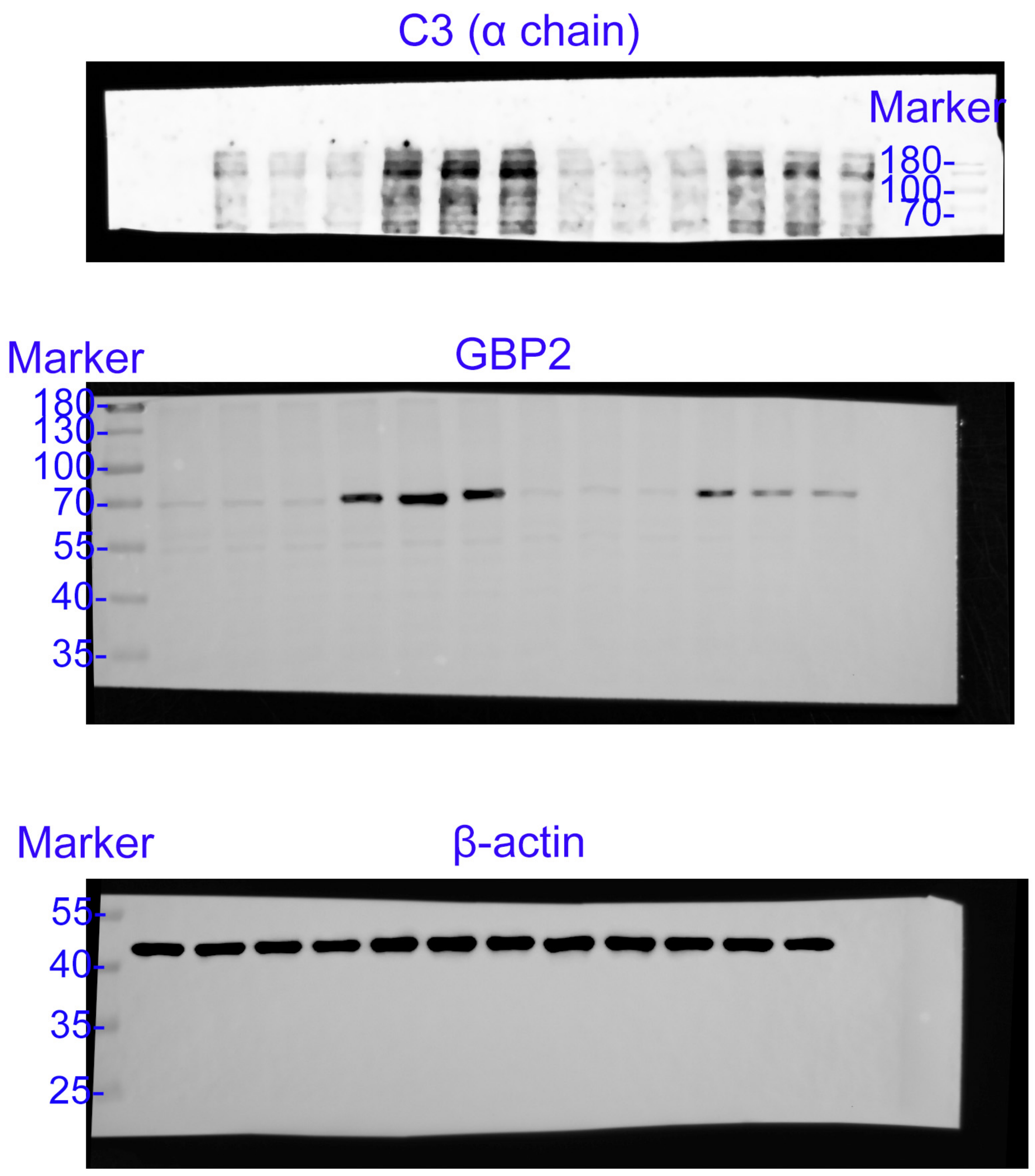

Figure 5F

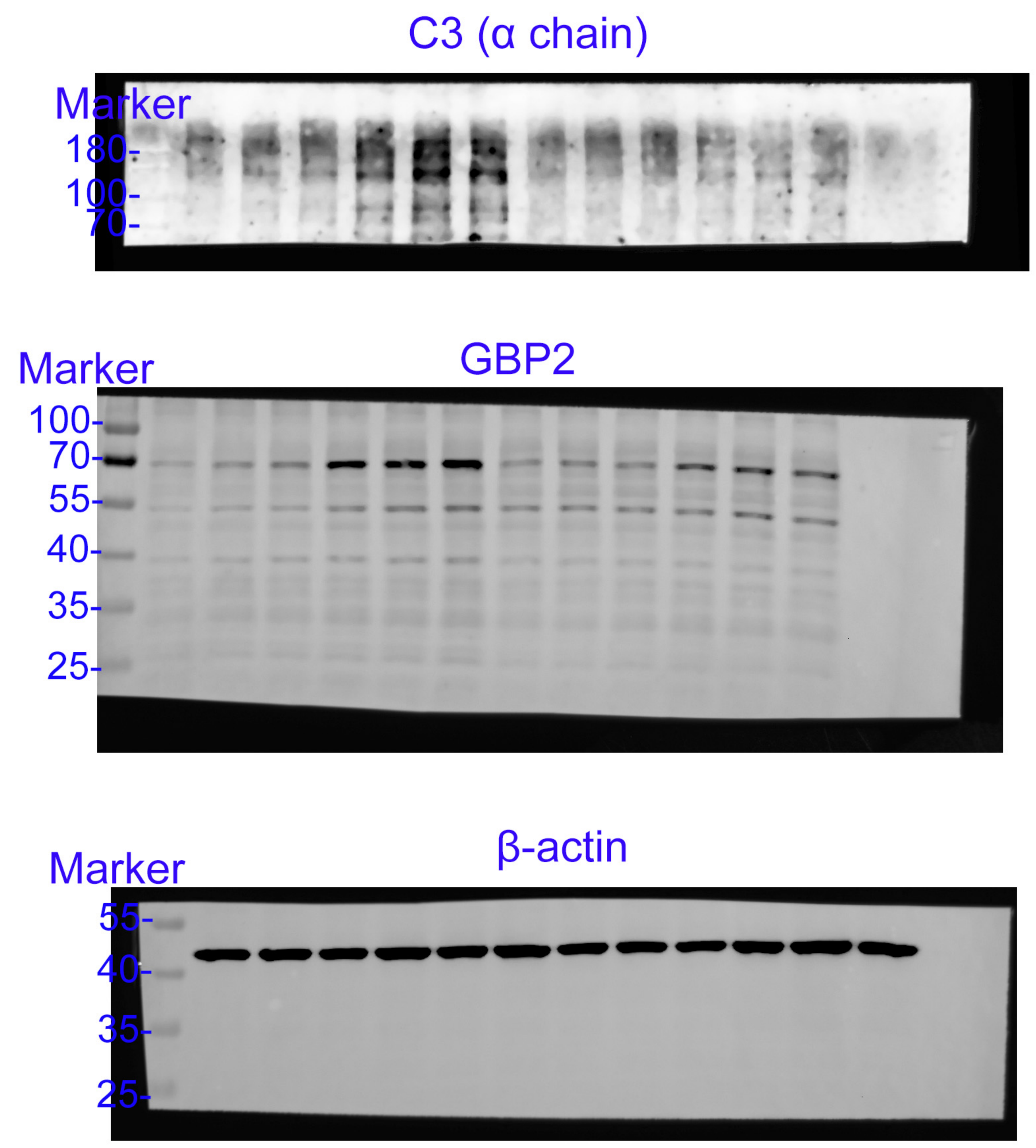

Figure 6

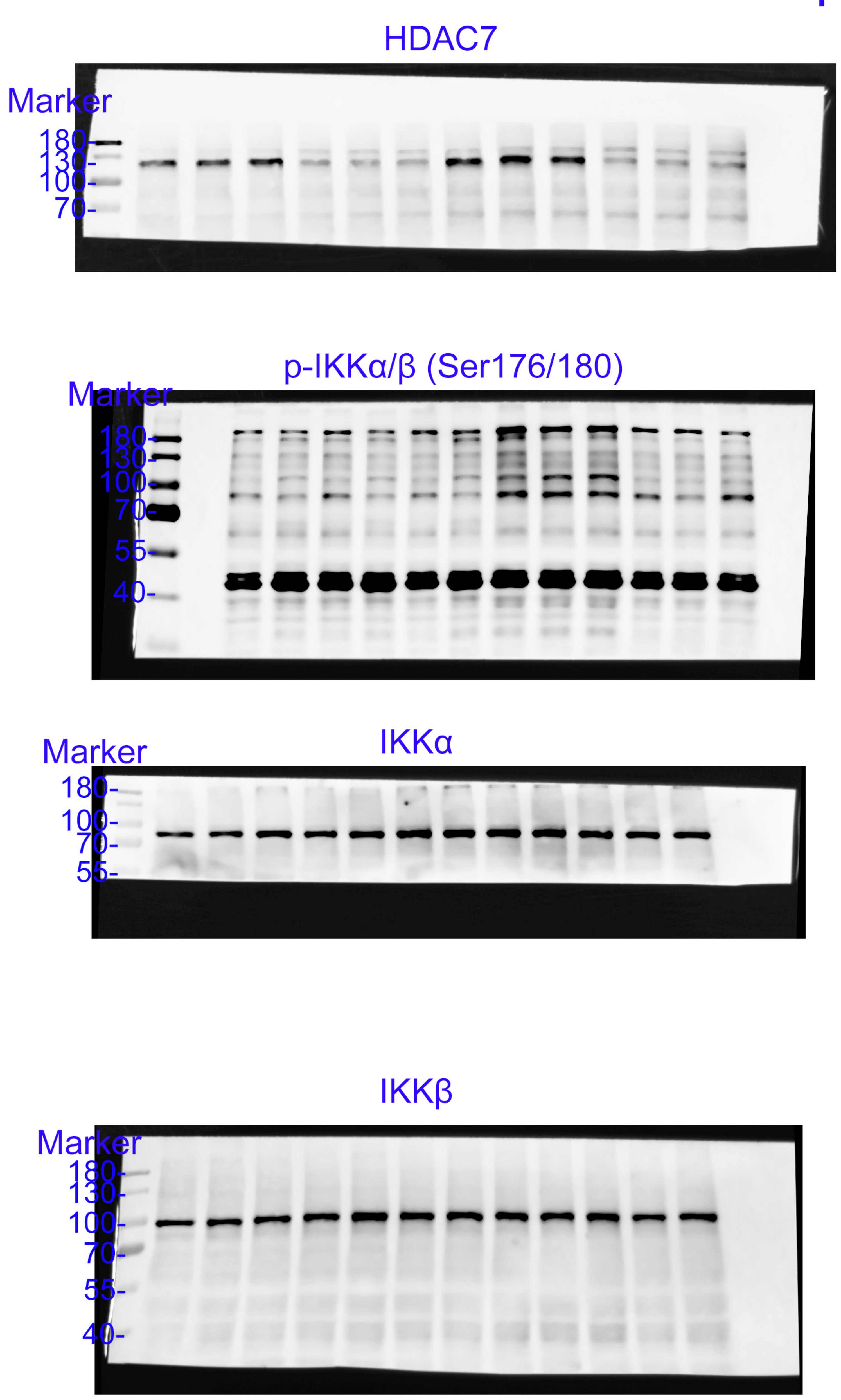

Figure 6C

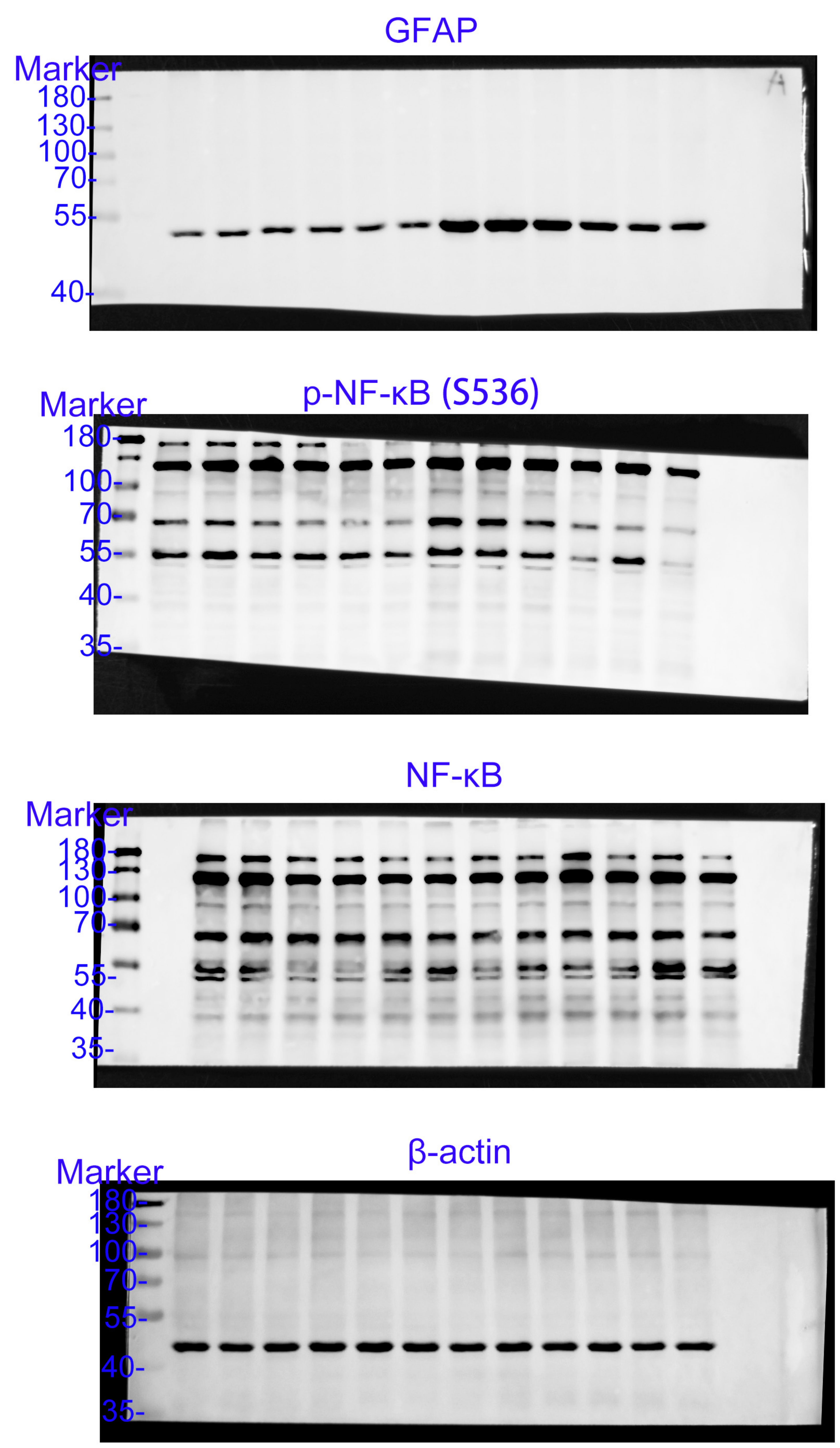

Figure 7

Figure 7B

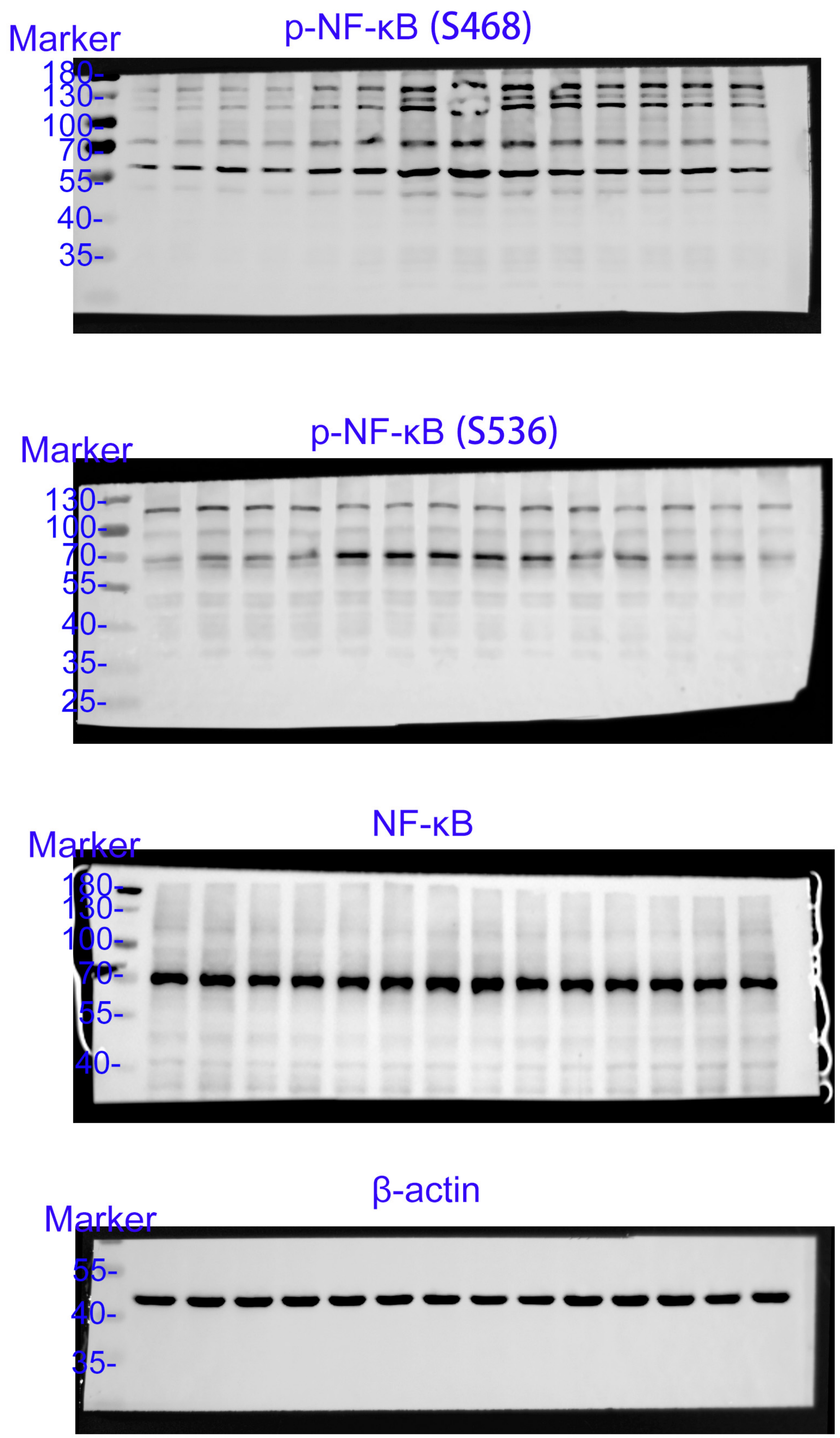

Figure 8

Figure 8B

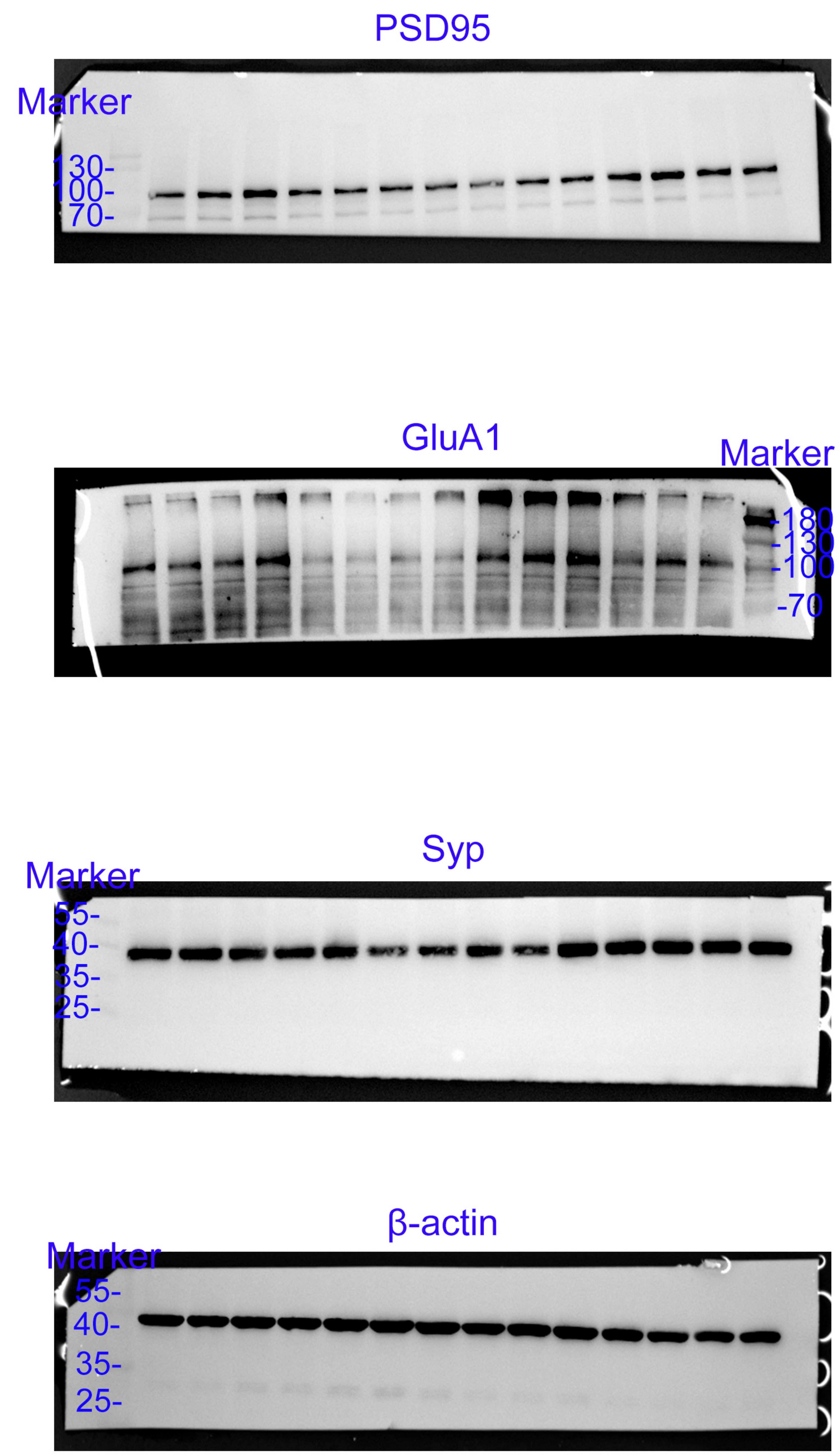

Supplement: Supplementary file 1 [file 13195_2026_2121_MOESM3_ESM.pdf]
