# Supplementary material for: HDAC7 acts as an astrocytic mediator of Aβ pathology that directly engages IKK to drive astrocyte neurotoxicity and neurodegeneration in Alzheimer’s disease
Source: Alzheimers Res Ther. 2026 Jun 22;18:196. doi: 10.1186/s13195-026-02121-5 (PMC13540850; doi:10.1186/s13195-026-02121-5)
Supplement: Supplementary file 2 [file 13195_2026_2121_MOESM2_ESM.docx]

**Supplementary figures**

**Supplementary Figure S1 Effects of astrocytic HDAC7 overexpression on Aβ pathology in APP/PS1 mice.**

(**A**) Representative immunohistochemistry staining images with anti-6E10 antibody in the hippocampus of APP/PS1 mice. (**B**) quantification of 6E10-positive plaque load area in the hippocampus of APP/PS1 mice. n = 6 mice per group. (**C**) ELISA analysis of soluble and insoluble Aβ_40_ and Aβ_42_ in the hippocampal tissues from APP/PS1 mice. Statistical significance was determined by unpaired Student’s t test. Data are shown as mean ± SEM, **p* < 0.05, ***p* < 0.01, ****p* < 0.001.

**Supplementary Figure S2 Expression pattern of astrocyte substate marker genes based on RNA-seq in WT and APP/PS1 mice** **with astrocytic HDAC7 overexpression.**

Heatmap showing the expression patterns of makers genes involving pan-reactive astrocyte, neurotoxic reactive astrocyte and neuroprotective astrocyte in the hippocampal tissues of WT and APP/PS1 mice with astrocytic HDAC7 overexpression.

**Supplementary Figure S3 Effects of resting astrocyte‑conditioned medium on neuronal viability.**

Calcein AM/EthD-1 staining showing cell viability (green: live cells) and death (megenta: dead cells) of primary neurons treated with non‑conditioned medium (Non-CM), conditioned medium from vehicle-treated wildtype astrocytes (CM‑veh), and conditioned medium from vector-expressing wildtype astrocytes (CM‑Vec). n = 6 dishes per group. Statistical significance was determined by one-way ANOVA with Tukey’s post hoc analysis. Data are shown as mean ± SEM, n. s, not significant.

 **Supplementary Figure S4 Effects of astrocytic HDAC7 knockdown on the expression levels of Il-1α, Tnfα and C1q, and Aβ pathology in APP/PS1 mice.**

(**A**) Representative immunostaining images and quantification of HDAC7 knockdown efficiency in the hippocampus of APP/PS1 mice after AAV injection, n = 6 mice per group. (**B**) RT-qPCR analysis of Il-1α, Tnfα and C1q in the hippocampus of WT and APP/PS1 mice with astrocytic HDAC7 knockdown, n = 6 mice per group. (**C**) Immunostaining analysis of Aβ plaques using anti-6E10 antibody in the hippocampal region of AAV-injected APP/PS1 mice, n = 6 mice per group. (**D**) ELISA analysis of soluble and insoluble Aβ_40_ and Aβ_42_ in the hippocampal tissues from AAV-injected APP/PS1 mice, n = 8 mice per group. Statistical significance was determined by unpaired Student’s t test. Data are shown as mean ± SEM, **p* < 0.05, ***p* < 0.01, ****p* < 0.001.

**Supplementary Figure S5 The expression levels of Il-1α, Tnfα and C1q in WT and APP/PS1 mice treated with TMP195.**

RT-qPCR analysis of Il-1α, Tnfα and C1q in the hippocampus of WT and APP/PS1 mice treated with TMP195 or vehicle, n = 6 mice per group.
